# Multi-level regulation of hindgut homeostasis by volatile fatty acid administration in dairy goats: linking microbial metabolism to epithelial inflammation and barrier function

**DOI:** 10.1128/msystems.00116-25

**Published:** 2025-06-13

**Authors:** Yongkang Zhen, Ali Rahmat, Chong Zhang, Jiaqi Lin, Jianjun Ma, Yuhong Zhong, Mengzhi Wang

**Affiliations:** 1 Laboratory of Metabolic Manipulation of Herbivorous Animal Nutrition, College of Animal Science and Technology, Yangzhou University 614678 , Yangzhou, Jiangsu, China; 2 State Key Laboratory of Sheep Genetic Improvement and Healthy Production, Xinjiang Academy of Agricultural Reclamation Sciences, Shihezi, Xinjiang, China; Pontificia Universidad Catolica de Chile, Santiago, Santiago, Chile

**Keywords:** volatile fatty acids, dairy goats, hindgut, gut homeostasis, gut bacteria

## Abstract

This study provided a comprehensive exploration of the nutritional regulation of volatile fatty acids (VFAs) on hindgut microbial metabolism and epithelial homeostasis in dairy goats. Twenty-four goats were orally administered sodium acetate (SA) at 0.8 g/kg of body weight (BW), propionate (SP) at 0.8 g/kg of BW, butyrate (SB) at 0.5 g/kg of BW, or saline (CON) before morning feeding for 12 days (*n* = 6/group). Serum and hindgut epithelial tissues were collected to measure antioxidant capacity, inflammatory cytokines, and tight junctions. Cecal contents were collected for 16S rRNA sequencing and metabolome analysis, and colonic epithelial cells were harvested for transcriptome sequencing. The data demonstrated that VFAs positively affected hindgut homeostasis. SB reduced serum malondialdehyde levels (*P* = 0.042), while SA and SP increased intestinal interleukin-10 concentration compared with CON (*P* < 0.001). All three VFAs enhanced gut barrier functions by increasing tight junctions compared with CON (*P* < 0.05). The data revealed distinct bacterial abundances and diversities associated with VFA administration, with notable responders including *Rikenellaceae* dgA-11 gut group, *Christensenellaceae* R-7 group, and *Bacteroides*. Metabolome analysis indicated significant changes in metabolic processes, such as purine, arachidonic acid, tyrosine, and tryptophan metabolism. Transcriptome analysis showed that SA and SP influenced endocrine and digestive functions, metal ion homeostasis, and muscle development in the colonic epithelium, with specific immune response pathways enriched in SB. Correlation analysis suggested interactions between hindgut bacteria and derived metabolites and epithelial homeostasis. In short, our study suggested potential strategies for improving gut health and overall well-being in goats through dietary interventions.

**IMPORTANCE:**

The volatile fatty acids (VFAs), mainly produced by rumen microbiota, play an important role in ruminal metabolic functions and epithelial health, but their impact on the hindgut has received limited attention. Our study highlighted the significant role of VFAs in hindgut bacterial metabolism and homeostasis, providing novel insights into the role of VFAs in regulating hindgut metabolism and physiological homeostasis beyond the rumen.

## INTRODUCTION

In the modern livestock industry, there is an increasing emphasis on animal welfare and healthy production to meet the demand for high-quality animal products. Ruminant animals, including cattle, sheep, and goats, play important roles in food production and provide 16% of global protein and 8% of global energy consumption through milk and meat (1). Goat milk, characterized by its high digestibility due to concentrations of monounsaturated and polyunsaturated fatty acids, as well as minerals like zinc, iron, and magnesium (2, 3), offers superior qualities such as alkalinity, probiotic carrier ability, and buffering capacity compared to cow milk (4). Despite these benefits, goats contribute only 2.3% of global milk production, a fraction compared to 82.6% from cattle (1, 5).

Improving gastrointestinal tract (GIT) health in ruminants is crucial for enhancing milk yield and quality for human consumption. The rumen and hindgut, key components of the GIT, serve as primary sites for nutrient digestion and absorption by GIT microorganisms (6). Moreover, the epithelial lining of the GIT acts as a critical barrier, preventing harmful substances from entering the portal circulation (7, 8). While recent studies have extensively investigated the regulation of ruminal bacteria, their unique digestive functions, mechanisms of nutrient absorption through the ruminal epithelium, and their impacts on ruminal health and homeostasis (9, 10), the regulation of the hindgut has received less attention despite its essential role.

The hindgut is mainly for digesting starch and other fermentable substrates that are not fully degraded in the rumen (11, 12), providing approximately 10% of dietary energy through fermentation by the cecal microbiota (13). Significant differences in colonization patterns of the cecal microbiome compared to the rumen microbiome have been observed (8, 14). Additionally, the cecal microbiota influences gut immunity, inflammation, and intestinal barrier function through host-microbe interactions, which is crucial for health and efficient production (15). For instance, the hindgut microbiome affects host oxidative stress by influencing glutathione synthesis in postpartum dairy cows (16). Unlike the multilayered rumen epithelium, although the hindgut epithelium has only a single layer of epithelial cells, it modulates inflammation and establishes a barrier against pathogens through tight junctions (17, 18).

Volatile fatty acids (VFAs), primarily acetate, propionate, and butyrate, are essential products of rumen microbial fermentation (10). They serve as important energy sources, providing over 70% of the energy utilized by ruminants (19). Studies in the rumen have demonstrated that supplementing VFAs, particularly butyrate, enhances ruminal fermentation, stimulates the development of rumen papillae and epithelial cells (20, 21), and exhibits anti-inflammatory, antitumorigenic, and antimicrobial effects (22). Notably, the hindgut contributes approximately 12% of the VFAs available to the organism (23). However, the specific effects of VFAs on the cecal bacteria profiles, their metabolic processes, and their interaction with homeostasis and molecular functions in the gut epithelium remain unclear.

Our hypothesis was that different VFAs can regulate the succession of cecal bacteria, influence their metabolic processes, and engage in crosstalk with epithelial cells to promote hindgut development and maintain homeostasis in goats. To investigate this hypothesis, we employed a dairy goat model and administered the animals with sodium acetate (SA), propionate (SP), and butyrate (SB), with saline serving as the control group. Through this approach, we aimed to generate comprehensive data encompassing VFA concentrations of cecal contents, serum biochemistry and antioxidant capacity parameters, and epithelial inflammatory cytokines and tight junctions. Additionally, we analyzed the bacterial and metabolome profiles of cecal contents and performed transcriptome sequencing of the colonic epithelial tissue.

## MATERIALS AND METHODS

### Animal management and experimental design

This animal experiment was conducted using female Guanzhong dairy goats at the experimental farm of Yangzhou University in Jiangsu Province, China, from November to December 2019. A total of 24 dairy goats with a mean body weight (BW) of 47.44 ± 3.38 kg at 1.5 years old in the early-lactation period were selected for this study. All goats were maintained under the same farming conditions and were fed a standard diet containing 60% forage and 40% concentrate mix *ad libitum* for 14 days at 1.50 kg to ensure stable feeding. Goats were individually housed in separate pens and fed twice daily at 8:00 and 18:00, with *ad libitum* access to feed and water. The feed ingredients and chemical composition are shown in Table 1 on a dry matter (DM) basis. The feed ingredients were composed of 41% oat hay, 29.5% corn grain, 14.5% soybean meal, 10% wheat bran, and 5% limestone powder, CaH_2_PO_4_, NaCl, and premix. The content of nutrient components was composed of 15.56% crude protein (CP), 35.26% neutral detergent fiber (NDF), 19.63% acid detergent fiber (ADF), 2.89% ether extract (EE), 0.41% Ca, and 0.47% P. The diet was formulated to meet the current feeding recommendations with a digestible energy (DE) of 10.25 MJ/kg. During the adaptation period, goats were manually milked before each feeding time in their tie stalls twice a week, and the milk yield was recorded. Milk samples were analyzed for composition and quality at Yangda Kangyuan Dairy Co., Ltd. (Yangzhou, Jiangsu, China). The average milk yield of the selected goats was 1.45 ± 0.20 kg/day, with average milk fat and protein compositions of 3.28% ± 0.30% and 3.05% ± 0.21%, respectively. All goats were vaccinated and had no history of antimicrobial agent (antibiotics, antifungals, or antivirals) administration or clinical signs of diseases.

**TABLE 1:** Feed ingredients and chemical composition (DM basis, %)

Items	Diet
Ingredients (% of DM)	
Oat hay	41.00
Corn grain	29.50
Soybean meal	14.50
Wheat bran	10.00
Limestone powder	0.35
CaH_2_PO_4_	0.15
NaCl	0.50
Premix^*a*^	4.00
Chemical composition (% of DM)	
CP	15.56
NDF	35.26
ADF	19.63
EE	2.89
Ca	0.41
P	0.47
DE (MJ/kg)	10.25

^*a*^ The premix provided the following per kg of diet: VA, 200,000 IU; VD3, 70,000 IU; VE, 350 IU; Fe, 1.6 g; Cu, 1.7 g; Zn, 8.2 g; Mn, 2.5 g; and Se, 40 mg.

**Fig 1:**
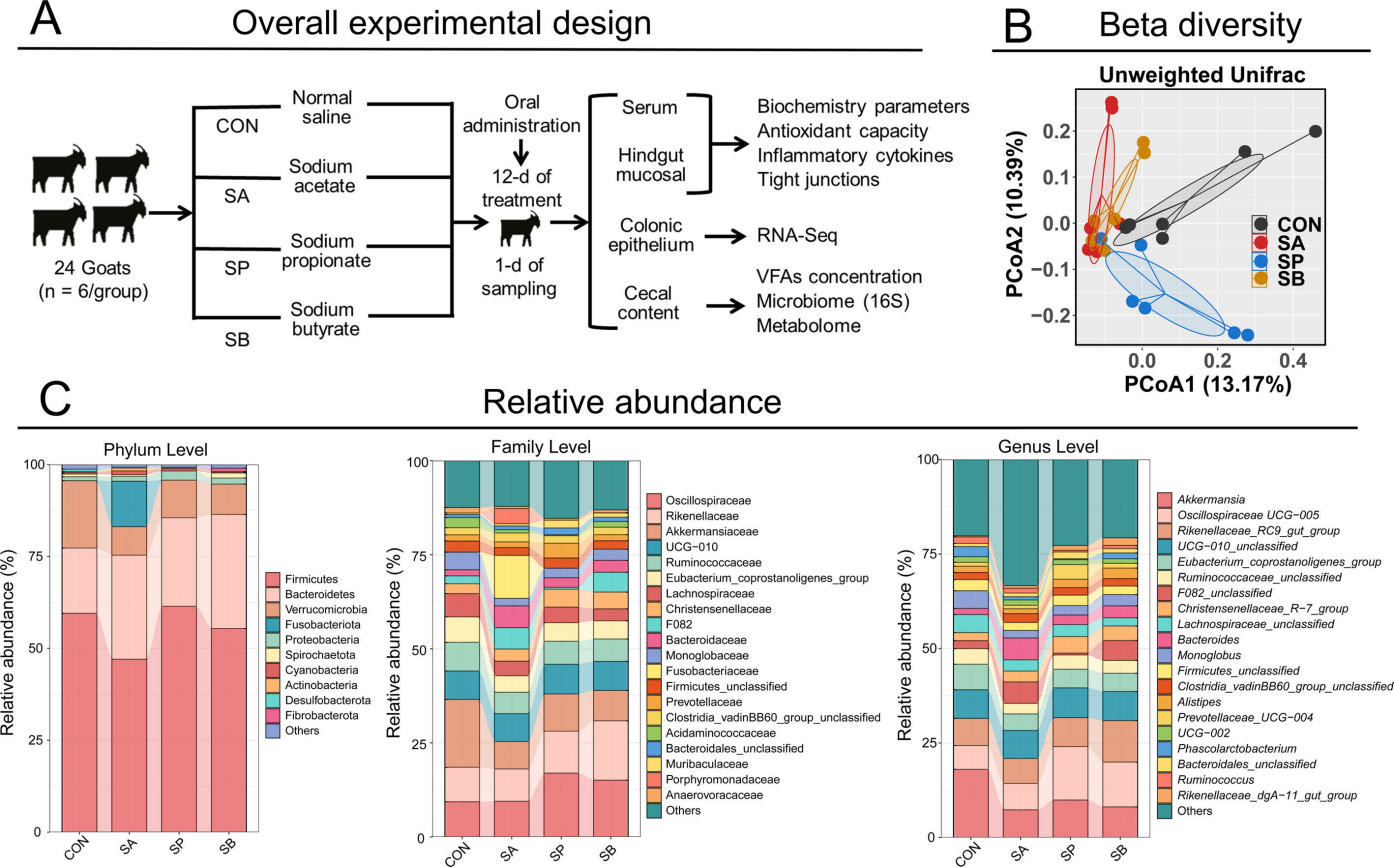
The schematic representation of experimental design, and the bacterial beta diversity and abundance in cecal contents. (**A**) The schematic representation of experimental design; (**B**) bacterial beta diversity using principal coordinate analysis (PCoA) plot calculated by unweighted UniFrac distances; (**C**) component proportions of cecal bacteria at phylum, family, and genus levels of four groups; *n* = 6 for 16S rRNA sequencing.

After adaptation, goats were randomly assigned to four groups (*n* = 6/group) to receive oral administration treatments. The BW and milk production were balanced among the groups, with no significant differences in these parameters. Goats were orally administered SA at 0.8 g/kg of BW per day (Sigma-Aldrich, USA, >99.0% purity), SP at 0.8 g/kg of BW per day (Sigma-Aldrich, >99.0% purity), SB at 0.5 g/kg of BW per day (Sigma-Aldrich, >98.0% purity), or normal saline (CON) at 1.0 L/day as the control group (Fig. 1A). The dosage of VFA administration solution was determined based on the optimal concentration selected according to our previous research and related studies in ruminants (24–27). Briefly, each goat was weighed, and the required amount of each VFA was calculated before daily morning feeding. The sodium powders were dissolved in 1.0 L of normal saline, and all solutions were calibrated to a consistent pH using a pH meter. Solutions were then orally administered through acid-resistant tubing with adjustable syringes 1 h before morning feeding, within 10 min per goat. The experiment lasted for 13 days, consisting of a 12-day administration period and a 1-day sampling period, which ensured that the animal management and dietary formulation were consistent with those in the adaptation period.

Analyses performed on feed samples were as follows: DM, according to AOAC official method 930.15 by drying them at 105°C for 2 h, and the ash content was determined by combustion at 550°C for 4 h (AOAC official method 942.05); CP, according to AOAC official method 990.02 by multiplying the nitrogen concentration by 6.25 using a Kjeltec Auto Analyzer (K9860, Jinan Hanon Instruments Co., Ltd, Jinan, Shandong, China); EE, according to AOAC official method 920.39 using a Soxtec Auto Analyzer (SOX500, Jinan Hanon Instruments Co., Ltd, Jinan, Shandong, China). The contents of NDF and ADF were determined according to the Van Soest method (28). The contents of Ca and P were measured according to AOAC official methods 968.08 and 965.17. DE was calculated from tabulated feed values according to NRC.

### Sample collection

All goats were fasted for 12 h on sampling day, and then the BW was recorded. Blood samples were collected from the coccygeal vein using procoagulant tubes and centrifuged at 3,000 × *g* for 15 min at 4°C to collect serum. Samples were then stored at −80°C for serum biochemistry and antioxidant capacity measurements. Subsequently, goats were anesthetized and euthanized by jugular vein puncture at the slaughterhouse of the experimental farm. The middle section of the cecum tissue was scraped using a microscope slide, and cecal contents were immediately collected and stored at −80°C for 16S rRNA sequencing and metabolome analysis. The entire colon, cecum, and rectal tissues were carefully separated, emptied of digesta, rinsed in phosphate-buffered saline (PBS), and weighed. Another 5.0 g of colonic and cecal epithelial tissues from the middle sections was harvested, rapidly frozen in liquid nitrogen, and stored at −80°C for measurements of inflammatory cytokines, tight junctions, and transcriptome sequencing analysis.

### Concentration of VFAs of cecal contents

The concentration of VFAs was determined using a gas chromatograph (GC-9A; Shimadzu, Kyoto, Japan). Briefly, 0.5 g of cecal content was diluted with 1.0 mL of distilled water. Then, 0.3 mL of metaphosphoric acid containing 20% of 60.0 mmol/L crotonic acid was added, vortexed, centrifuged, and filtered through a 0.25-μm-pore-size syringe filter. The supernatant was then collected for subsequent analyses. Portions (0.5 µL) of test samples and standard solution mix were run through a CP-WAX capillary column (length, 30.0 m; inner diameter, 0.53 mm; and film thickness, 1.0 mm) in a gas chromatograph. Program settings and calculations followed the method of our laboratory (27).

### Serum biochemistry and antioxidant capacity parameters

Serum concentrations of lactate dehydrogenase (LDH; BC0685), creatinine (CREA; BC4915), blood urea nitrogen (BUN; BC1535), triglyceride (TG; BC0625), cholesterol (CHOL; BC1985), high-density lipoprotein cholesterol (HDL-C; BC5325), and low-density lipoprotein cholesterol (LDL-C; BC5335) were measured using commercial kits (Beijing Solarbio Science & Technology Co., Ltd., Beijing, China) following the manufacturer’s protocols. For serum antioxidant capacity parameters, the total antioxidant capacity (T-AOC; BC1315), glutathione peroxidase (GSH-Px; BC1195), catalase (CAT; BC0205), superoxide dismutase (SOD; BC0170), and malondialdehyde (MDA; BC0025) were measured using commercial assay kits from Solarbio according to the manufacturer’s instructions.

### Concentration of inflammatory cytokines and tight junctions of hindgut epithelial tissue

To measure concentrations of total protein (TP), inflammatory cytokines, and tight junctions, 0.1 g of colonic and cecal epithelial tissue was homogenized with 1 mL PBS and then centrifuged at 13,400 × *g* at 4°C for 10 min to collect the supernatant. TP concentration was measured using a commercial assay kit (A045-2, Nanjing Jiancheng Bioengineering Institute, Nanjing, China). Concentrations of interleukin-1β (IL-1β), interleukin-6 (IL-6), interleukin-10 (IL-10), tumor necrosis factor α (TNF-α), Claudin1, occludin, and tight junction protein 1 (ZO-1) were measured using enzyme-linked immunosorbent assay (ELISA) with a multifunctional microplate reader (SpectraMax M5, Molecular Devices, Sunnyvale, CA, USA) following the manufacturer’s instructions (MLBio, Shanghai, China). Parameters were normalized by TP to determine concentrations per milligram protein.

### 16S rRNA sequencing and data processing of cecal contents

Total microbial DNA from cecal contents was extracted using FastPure Bacteria DNA Isolation Mini Kit (DC103, Vazyme Biotech Co., Ltd., Nanjing, China) for 16S rRNA sequencing. DNA quality was assessed by agarose gel electrophoresis and quantified using a UV spectrophotometer. High-throughput sequencing of the V3-V4 region of the 16S rRNA gene was performed using an Illumina NovaSeq PE250 (Illumina, San Diego, CA, USA) at LC-Bio Technology Co., Ltd. (Hangzhou, Zhejiang, China). The V3-V4 region was amplified with primers 341F (5′-CCTACGGGNGGCWGCAG-3′) and 805R (5′-GACTACHVGGGTATCTAATCC-3′) (29).

For data processing, paired-end reads were merged using FLASH software (version 1.2.7) (30). Quality filtering was then performed under specific filtering conditions to obtain the high-quality clean reads using Fqtrim software (version 0.9.4). Chimeric sequences were filtered using VSEARCH software (version 2.3.4) (31). The feature table and feature sequence were obtained after dereplication using DADA2 software (version 1.10.1) (32). Bacterial alpha diversity (Chao 1, Shannon, and Simpson) was used to assess the complexity of species diversity in QIIME2 software (version 2019.4) (33). Beta diversity was calculated using principal coordinate analysis (PCoA) based on Unweighted UniFrac distance (34). The amplicon sequence variant (ASV) profiling analysis and microbial relative abundance analysis were performed using R software (version 4.2). Given that the microbial alpha diversity parameters and the relative abundances of bacterial phyla and genera were not normally distributed, the Wilcoxon rank-sum test was used to analyze the differences between the treatments. The significant taxonomic differences in response to VFA administration at genus classification were also selected using linear discriminant analysis effect size (LEfSe) analysis (LDA score > 3) (35).

### LC-MS metabolome and data processing of cecal contents

An accurately weighed 0.1 g sample of cecal contents was used for LC-MS metabolome analysis. Metabolome analysis was performed on a Vanquish UHPLC System (ThermoFisher Scientific, Santa Clara, CA, USA) using an ACQUITY UPLC HSS T3 column (150 mm × 2.1 mm, 1.8 µm) (Waters, Milford, MA, USA) at a column temperature of 40°C, a flow rate of 0.25 mL/min, and an injection volume of 2 µL. The LC-ESI (+)-MS and LC-ESI (−)-MS analyses followed a previously described protocol (36). Then, mass spectrometric detection of metabolites was also performed on a Q Exactive HF-X (Thermo Fisher Scientific, USA) with an ESI ion source. The parameters were set up following a previous study (37). Metabolome analysis was performed at Panomix Biomedical Tech Co., Ltd. (Suzhou, Jiangsu, China).

For data processing, the raw data were converted to mzXML format by MSConvert using ProteoWizard software (version 3.0.8789). Data were then processed using XCMS for feature detection, retention time correction, and alignment (38, 39). The metabolites were identified by accurate mass (<30 ppm) and MS/MS data, which were matched using HMDB, MassBank, LipidMaps, mzCloud, and KEGG databases. Then, normalized data were imported into SIMCA software (version 14.1) (AB Umetrics, Umea, Sweden) and preprocessed using PAR scaling and mean centering before principal components analysis (PCA) (40). The orthogonal partial least squares discrimination analysis (OPLS-DA) was performed to allow the determination of discriminating metabolites using the variable importance on projection (VIP) value. The OPLS-DA model was tested for overfitting with 200 permutation tests. Then, nonparametric tests were performed on non-normally distributed metabolomic data using the Wilcoxon-Mann-Whitney test to calculate the *P-*value. The *P*-value, VIP, and fold change (FC) were applied to discover the contributable variable for classification. Finally, *P*-value < 0.05 and VIP > 1 were considered to be statistically significant metabolites. The functional enrichment analysis was performed using the KEGG database at the MetaboAnalyst 5.0 website (41).

### Transcriptome sequencing and data processing of colonic epithelial tissue

Briefly, total RNA of colonic epithelial tissue sample of each goat was isolated and purified using FastPure Cell/Tissue Total RNA Isolation Kit V2 (RC112, Vazyme Biotech Co., Ltd., Nanjing, China) for transcriptome sequencing analysis. The RNA integrity was assessed by Agilent 2100 with RIN number > 7.0. The cDNA library was constructed and then sequenced on the DNBSEQ-T7 sequencer platform (BGI, Shenzhen, China) at Novogene Co., Ltd. (Beijing, China).

For data processing, the Fastp software (version 0.23.1) (42) was used to perform quality control on the raw data and obtain clean data. Then, the HISAT2 software (version 2.1.0) (43) was used to align the obtained clean data to the latest reference genome of goats (*Capra hircus*, ARS1.2) (44). The Samtools software (version 1.10) was used to sort and convert the SAM files to BAM format (45), while the Stringtie software (version 2.2.1) was used to assemble and quantify the genes based on read counts (43). The PCA was then generated in R software (version 4.2). Finally, the identification of differentially expressed genes (DEGs) was estimated using DESeq2 software (version 1.36.0) based on the normalized gene count data. DEGs were identified using a threshold of *P* < 0.05 and |log2 fold change| > 1. Then, a Venn plot, a volcano plot, and a dot plot of all DEGs were generated. Finally, the functional enrichment analysis was performed using KEGG and GO databases.

### Statistical analysis and visualization

The Statistical Package for Social Sciences (SPSS 25.0, SPSS, Inc., Chicago, IL, USA) software was used for the statistical analyses except for the multi-omics data. The data were fitted into a general linear model for a completely randomized design. The different VFAs and saline treatments were considered as the main factor, while the animals were considered as a random factor. The normality of data distribution was verified by the Kolmogorov-Smirnov test. The comparison of the differences of the normally distributed data, such as BW, hindgut weight, VFA concentrations, serum biochemistry and antioxidant capacity parameters, inflammatory cytokines, and tight junctions, was subjected to the one-way analysis of variance (one-way ANOVA) and *post hoc* Duncan’s test. Significant and extremely significant differences were declared at *P* < 0.05 and *P* < 0.01, respectively. Correlation analysis between selected parameters and multi-omics data was calculated using Pearson’s or Spearman correlation coefficients and Mantel’s test. R package ggplot2, Pheatmap, ggcor, and GraphPad Prism 6.0 software (GraphPad, California, USA) were used for graphics.

## RESULTS

### Body weight, hindgut weight, and the concentration of VFAs in cecal contents

The BW, hindgut weight, and concentration of VFAs in cecal contents are presented in Table 2. Oral administration of the three VFAs and saline did not impact the BW of all goats (*P* = 0.089) but significantly affected hindgut weight. Specifically, SA and SB significantly increased cecal weight compared with CON and SP (*P* < 0.001). Additionally, SA increased rectal weight compared with the other three groups (*P* < 0.001). However, VFA administration did not affect colonic weight (*P* = 0.125). Regarding the concentration of VFAs in cecal contents, SA significantly increased the concentration of total VFAs (*P* = 0.017), acetate (*P* = 0.005), and other VFAs (valerate, isobutyrate, and isovalerate) (*P* = 0.008) compared with the other three groups. SB also significantly increased the concentration of butyrate compared with CON and SP (*P* = 0.024). However, the concentration of propionate did not differ significantly among the four groups (*P* = 0.585).

**TABLE 2:** Body weight, hindgut weight, and the concentration of VFAs in colonic and cecal contents^*a*^

Items	CON	SA	SP	SB	SEM	*P*-value
Body weight and hindgut weight						
Body weight (kg)	48.77	48.83	51.11	50.69	0.373	0.089
Colonic weight (g)	146.52	146.13	130.05	126.64	3.825	0.125
Cecal weight (g)	239.23b	296.14a	224.62b	270.50a	6.612	<0.001
Rectal weight (g)	182.20b	208.98a	168.43b	167.02b	3.856	<0.001
Concentration of VFAs in cecal contents						
Total VFAs (μmol/g)	31.64b	40.38a	30.61b	34.03b	1.281	0.017
Acetate (μmol/g)	23.69b	30.75a	22.81b	24.49b	1.071	0.005
Propionate (μmol/g)	5.45	5.96	5.22	5.25	0.205	0.585
Butyrate (μmol/g)	1.34b	1.87ab	1.31b	2.85a	0.223	0.024
Other VFAs^*b*^ (μmol/g)	1.16b	1.81a	1.27b	1.44b	0.081	0.008

^*a*^ Values with different letters within a row mean statistically significant (*P* < 0.05 or *P* < 0.01).

^*b*^ Other VFAs included valerate, isobutyrate, and isovalerate.

### Serum biochemistry and antioxidant capacity parameters

The serum biochemistry and antioxidant capacity parameters are shown in Table 3. SP increased the concentration of LDH compared with the other three groups (*P* < 0.001), while both SA and SP increased the concentration of CREA compared with CON and SB (*P* = 0.003). No significant difference was observed among the four groups for other serum parameters, including BUN (*P* = 0.405), TG (*P* = 0.230), CHOL (*P* = 0.207), HDL-C (*P* = 0.602), and LDL-C (*P* = 0.340). For serum antioxidant capacity parameters, VFA administration did not impact serum T-AOC (*P* = 0.379), GSH-Px (*P* = 0.513), and CAT (*P* = 0.417) compared with CON. However, the concentration of SOD was significantly decreased in SA and SP compared with CON and SB (*P* = 0.038), and SB also decreased the concentration of MDA compared with SP (*P* = 0.042).

**TABLE 3:** Serum biochemistry and antioxidant capacity parameters^*a*^

Items	CON	SA	SP	SB	SEM	*P*-value
Serum biochemistry parameters						
LDH (U/L)	235.33b	242.44b	370.06a	234.44b	13.646	<0.001
CREA (μmol/L)	95.83b	107.08a	108.14a	98.63b	1.576	0.003
BUN (mmol/L)	4.69	4.56	3.93	4.66	0.178	0.405
TG (mmol/L)	0.08	0.07	0.06	0.09	0.005	0.230
CHOL (mmol/L)	2.08	1.62	1.73	1.91	0.081	0.207
HDL-C (mmol/L)	1.38	1.18	1.38	1.43	0.066	0.602
LDL-C (mmol/L)	0.58	0.48	0.50	0.57	0.024	0.340
Serum antioxidant capacity parameters						
T-AOC (μmol/mL)	1.29	1.17	1.31	1.09	0.049	0.379
GSH-Px (U/mL)	240.11	203.80	260.88	215.48	14.145	0.513
CAT (U/mL)	5.78	4.20	4.99	4.37	0.358	0.417
SOD (U/mL)	9.16a	6.26b	6.72b	7.39ab	0.397	0.038
MDA (nmol/mL)	0.65ab	0.66ab	0.85a	0.21b	0.085	0.042

^*a*^ Values with different letters within a row mean statistically significant (*P* < 0.05 or *P* < 0.01).

### Cecal bacterial structure and selection of significant taxonomic differences

**Fig 2:**
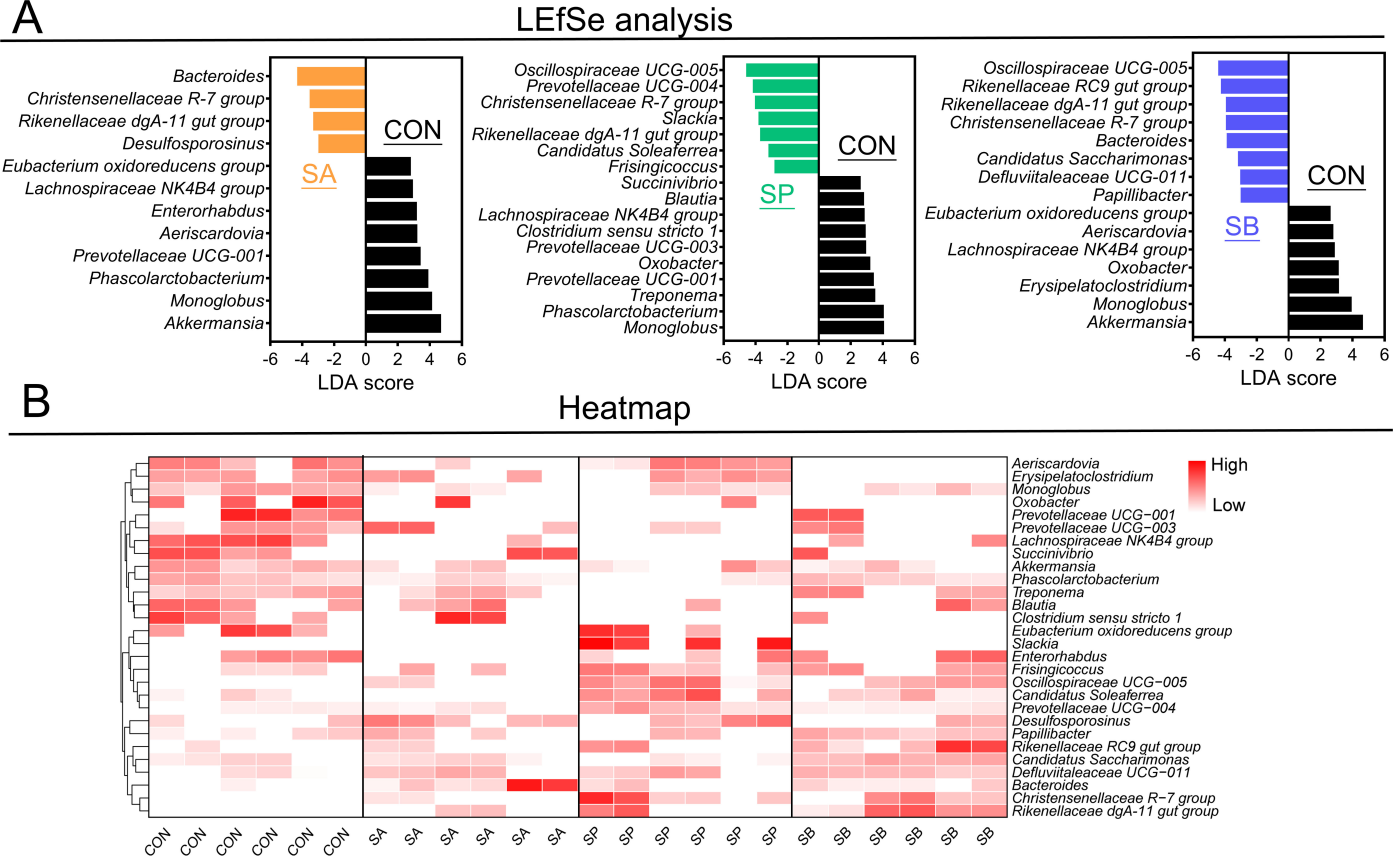
Selection of candidate cecal bacteria in response to VFA administration and different bacterial taxonomy. (**A**) LEfSe analysis of candidate cecal bacteria and different bacterial taxonomy in response to VFA administration at the genus level, only LDA score greater than 3 was marked; (**B**) cluster heatmap of candidate cecal bacteria of four groups.

Table 4 and Fig. 2 illustrate the hindgut bacterial patterns and alterations in their diversity and taxonomy of cecal contents. Regarding alpha diversity indexes (Table 4), SA significantly decreased the Chao 1 index compared with CON and SP (*P* = 0.016); however, no significant difference was found in the Shannon (*P* = 0.367) and Simpson (*P* = 0.373) indexes among the four groups. Bacterial beta diversity, calculated using the PCoA diagram based on unweighted UniFrac distance, revealed milder separation among the four groups (Fig. 1B), indicating that VFA administration influenced cecal bacterial succession patterns and community structure. At the phylum level, 10 phyla were identified, with the dominant bacterial phyla including *Firmicutes*, *Bacteroidetes*, *Verrucomicrobia, Proteobacteria,* etc. (Fig. 1C). The dominant bacterial families were *Oscillospiraceae*, *Rikenellaceae*, *Akkermansiaceae*, UCG-010, *Ruminococcaceae*, *Eubacterium coprostanoligenes* group, *Lachnospiraceae*, *Christensenellaceae,* etc. (Fig. 1C). The dominant bacterial genera included *Akkermansia*, *Oscillospiraceae* UCG-005, *Rikenellaceae* RC9 gut group, UCG-010 unclassified, *Eubacterium coprostanoligenes* group, *Christensenellaceae* R-7 group*,* etc. (Fig. 1C).

**TABLE 4:** Bacterial alpha diversity indexes in cecal content^*a*^

Items	CON	SA	SP	SB	SEM	*P*-value
Chao 1 index	493.30a	286.00b	494.36a	346.08ab	30.239	0.016
Shannon index	7.42	6.61	7.45	7.29	0.188	0.367
Simpson index	0.97	0.96	0.98	0.98	0.006	0.373

^*a*^ Values with different letters within a row mean statistically significant (*P* < 0.05 or *P* < 0.01).

The LEfSe method was used to identify candidate bacteria in response to VFA administration and significant bacterial taxonomy differences (Fig. 2). Compared with CON, SA significantly decreased *Akkermansia*, *Monoglobus*, *Phascolarctobacterium*, *Prevotellaceae* UCG-001, *Aeriscardovia*, *Enterorhabdus*, *Lachnospiraceae* NK4B4 group, and *Eubacterium oxidoreducens* group while significantly increasing *Bacteroides, Christensenellaceae* R-7 group, *Rikenellaceae* dgA-11 gut group, and *Desulfosporosinus* (Fig. 2A). Then, *Oscillospiraceae* UCG-005*, Prevotellaceae* UCG-004, *Christensenellaceae* R-7 group, *Slackia*, *Rikenellaceae* dgA-11 gut group, *Candidatus* Soleaferrea, and *Frisingicoccus* were enriched in SP, while *Monoglobus*, *Phascolarctobacterium*, *Treponema*, *Prevotellaceae* UCG-001, *Oxobacter*, *Prevotellaceae* UCG-003, *Clostridium sensu stricto* 1, *Lachnospiraceae* NK4B4 group, *Blautia*, and *Succinivibrio* were enriched in CON (Fig. 2A). Finally, SB decreased *Akkermansia*, *Monoglobus*, *Erysipelatoclostridium*, *Oxobacter*, *Lachnospiraceae* NK4B4 group, *Aeriscardovia*, and *Eubacterium oxidoreducens* group while increasing *Oscillospiraceae* UCG-005*, Rikenellaceae* RC9 gut group, *Rikenellaceae* dgA-11 gut group, *Christensenellaceae* R-7 group, *Bacteroides*, *Candidatus* Saccharimonas, *Defluviitaleaceae* UCG-011, and *Papillibacter* compared with CON (Fig. 2A). The cluster heatmap visualized in Fig. 2B shows the higher relative abundance of significant taxonomic differences at the bacterial genus level in each group compared to others.

### Cecal metabolome profiling

**Fig 3:**
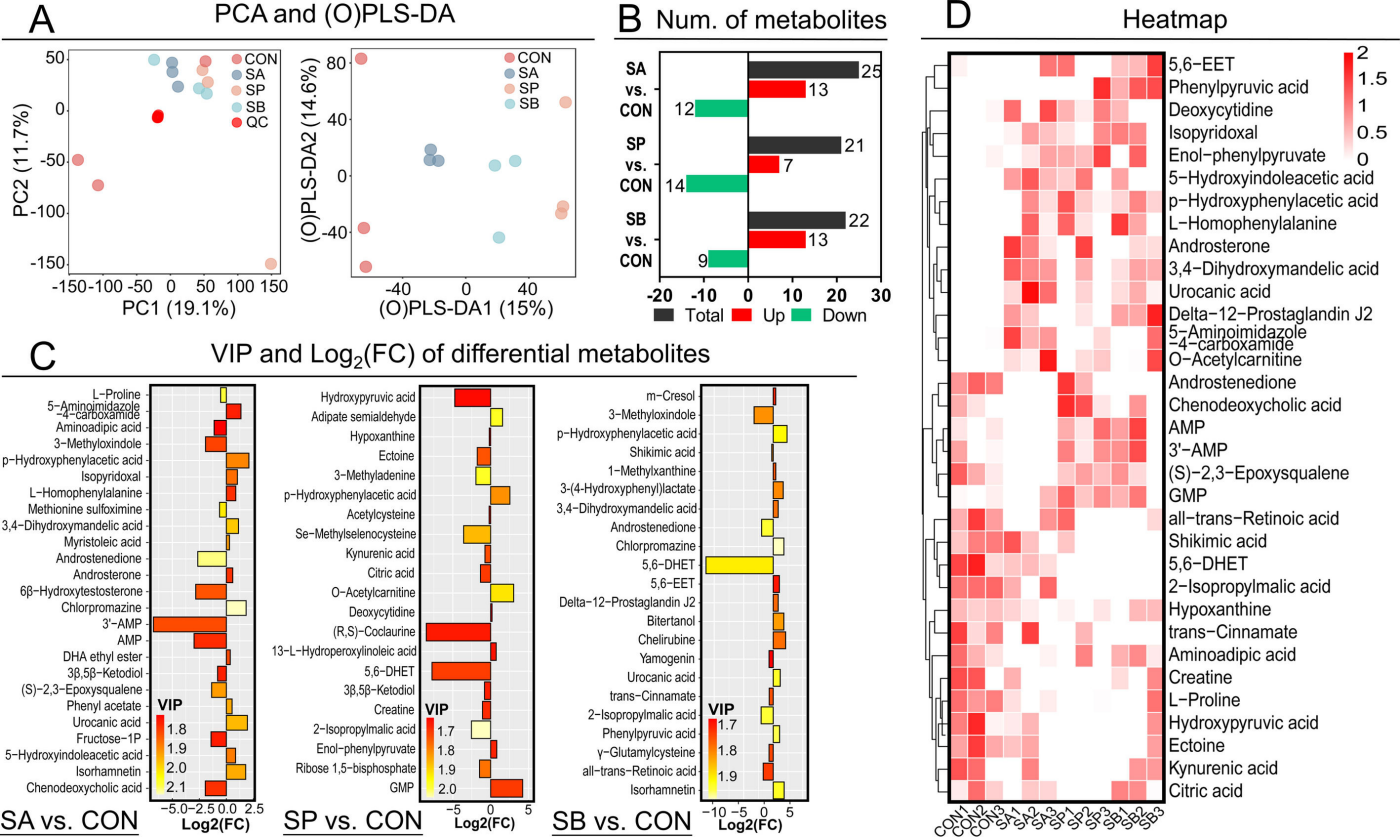
The LC-MS metabolome profiles in cecal contents and selection of differential metabolites. (**A**) The distributions of all metabolites using PCA and OPLS-DA score plots of groups and QC samples; (**B**) the number of differential metabolites between each VFA and CON in cecal contents; (**C**) the VIP and log_2_(FC) of differential metabolites; (**D**) the cluster heatmap of differential metabolites of four groups in cecal contents; *n* = 3 for LC-MS metabolome analysis.

Cecal metabolome analysis using LC-MS was performed to study microbial metabolism profiling in response to VFA administration (Fig. 3). A total of 410 small metabolites were identified among the four groups and QC samples. The PCA score plot indicated no separation among the groups and QC samples (Fig. 3A). However, the OPLS-DA plot demonstrated that metabolic patterns in cecal contents were significantly separated among the groups, indicating that VFA administration significantly altered microbial metabolism patterns in the hindgut (Fig. 3A).

**Fig 4:**
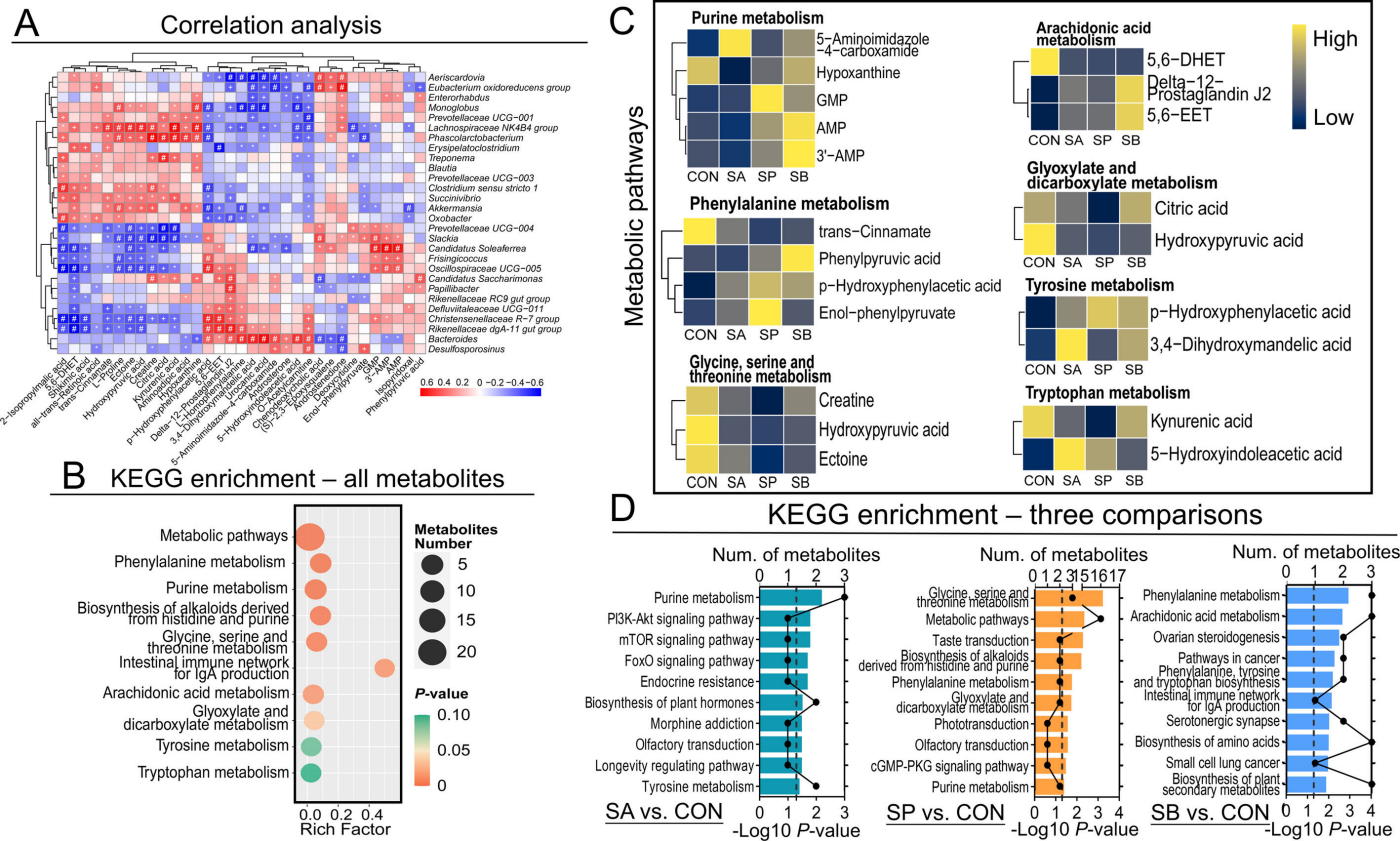
Correlation analysis between differential metabolites and candidate bacteria, and functional enrichment analysis of differential metabolites. (**A**) Correlation analysis between differential metabolites and candidate bacteria. The analysis is based on the Spearman correlation coefficient. *P < 0.05; ^+^*P* < 0.01; and ^#^*P* < 0.001; (**B**) functional enrichment analysis of all differential metabolites using KEGG database of four groups; (**C**) heatmaps of the abundances of differential metabolites in metabolic pathways of four groups; (**D**) functional enrichment analysis of differential metabolites of each comparison using KEGG database.

Metabolites with VIP > 1 and *P* < 0.05 were considered significantly different. In SA, 12 metabolites, including L-proline, aminoadipic acid, 3′-AMP, AMP, and chenodeoxycholic acid, were significantly decreased, while 13 metabolites, including isopyridoxal, L-homophenylalanine, androsterone, chlorpromazine, and urocanic acid, were significantly higher than those in CON (Table S1; Fig. 3B and C). In SP, 14 metabolites were decreased, including hydroxypyruvic acid, ectoine, citric acid, 5,6-DHET, and creatine, while seven metabolites, including adipate semialdehyde, p-hydroxyphenylacetic acid, O-acetylcarnitine, deoxycytidine, and GMP, were increased compared with CON (Table S1; Fig. 3B and C). In SB, nine metabolites were decreased, including 3-methyloxindole, shikimic acid, androstenedione, 5,6-DHET, and 2-isopropylmalic acid, while 13 metabolites, including 3,4-dihydroxymandelic acid, bitertanol, chelirubine, urocanic acid, and phenylpyruvic acid, were increased compared with CON (Table S1; Fig. 3B and C). The cluster heatmap identified the abundances of selected differential metabolites (Fig. 3D). A correlation heatmap showed significant correlations between differential bacteria and metabolites, suggesting that alterations in hindgut metabolic patterns induced by VFA administration may be mediated by gut microbes (Fig. 4A).

### Functional enrichment analysis of differential metabolites

Pathway enrichment analysis of cecal differential metabolites was performed (Fig. 4). KEGG enrichment analysis of all differential metabolites after VFA administration revealed significant impacts on several metabolic pathways (Fig. 4B). Differential metabolites were enriched in metabolic pathways, including purine metabolism (AMP, GMP, hypoxanthine, 3′-AMP, and 5-aminoimidazole-4-carboxamide); phenylalanine metabolism (phenylpyruvic acid, *trans*-cinnamate, p-hydroxyphenylacetic acid, and enol-phenylpyruvate); glycine, serine, and threonine metabolism (hydroxypyruvic acid, creatine, and ectoine); arachidonic acid metabolism (delta-12-prostaglandin J2, 5,6-EET, and 5,6-DHET); glyoxylate and dicarboxylate metabolism (citric acid and hydroxypyruvic acid); tyrosine metabolism (p-hydroxyphenylacetic acid and 3,4-dihydroxymandelic acid); and tryptophan metabolism pathway (kynurenic acid and 5-hydroxyindoleacetic acid).

Functional enrichment analysis also revealed that, except for metabolic pathways, differential metabolites may also be enriched in other functional pathways. Differential metabolites were enriched in the longevity-regulating pathway, endocrine resistance, mTOR signaling pathway, and PI3K-Akt signaling pathway between SA and CON. Metabolites were enriched in the cGMP-PKG signaling pathway, olfactory transduction, and taste transduction between SP and CON. Most differential metabolites were enriched in biosynthesis of amino acids, serotonergic synapse, and intestinal immune network for IgA production between SB and CON (Fig. 4D).

### Colonic epithelial transcriptome sequencing profiling

**Fig 5:**
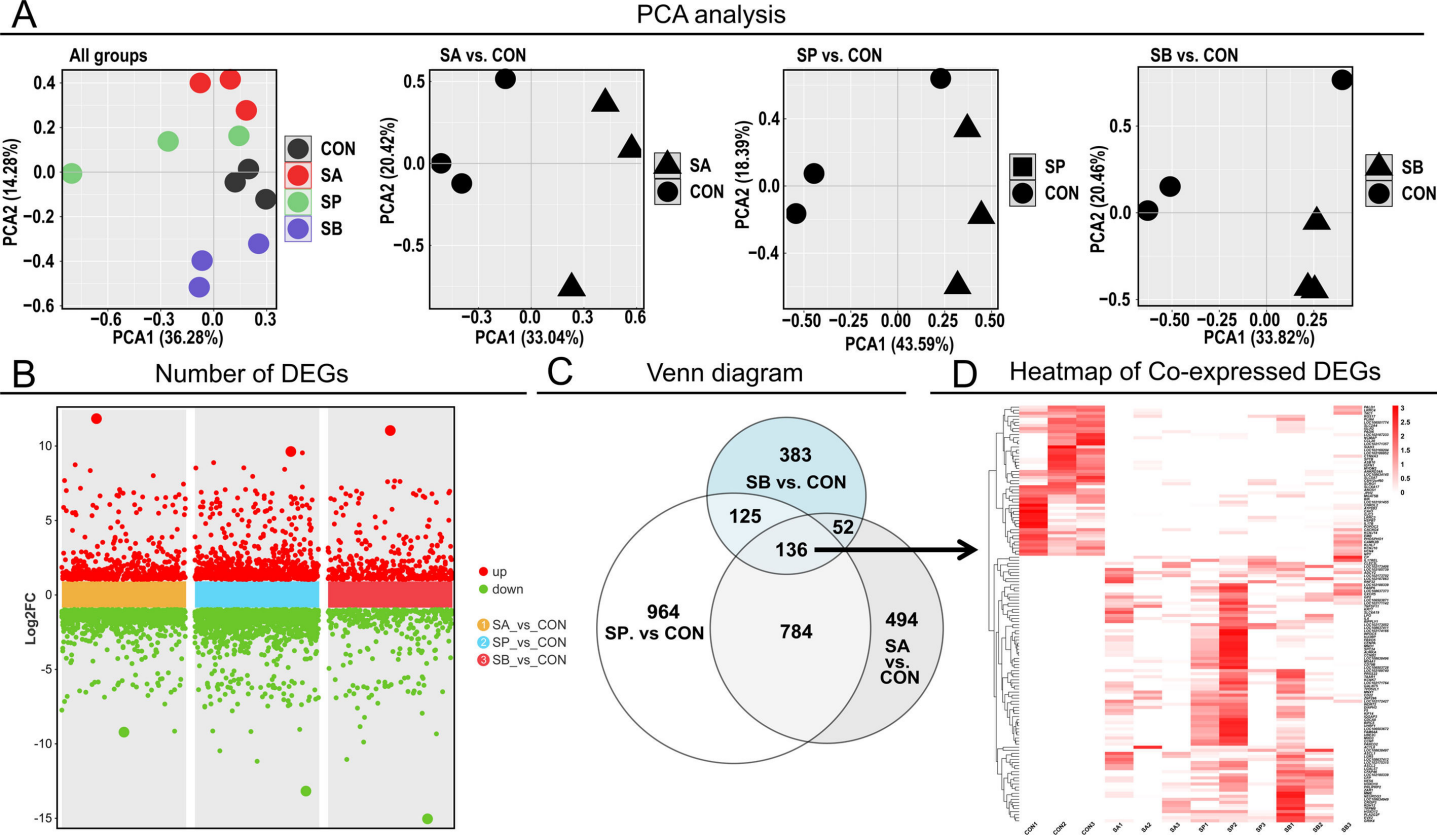
The transcriptome sequencing profiles in colonic epithelium. (**A**) The PCA using a combined data set of all genes of four groups; (**B**) the number of up- and downregulated DEGs between each VFA and CON; (**C**) Venn diagram of the number of single- and co-expressed DEGs between each VFA and CON; (**D**) heatmap of the expression level of the co-expressed DEGs in four groups; *n* = 3 for transcriptome sequencing analysis.

We performed transcriptome sequencing of colonic epithelium samples to study the effect of microbiota and derived metabolites on epithelial metabolism, absorption, and signal transduction. The PCA using the combined data set of all genes indicated significant separation among the four groups (Fig. 5A). Specifically, PCA of the SA, SP, and SB groups showed marked separation from the CON group (Fig. 5A). The expression level of DEGs was further analyzed using a cutoff of *P* < 0.05. The number of DEGs between each VFA and CON is shown in Fig. 5B, with detailed information in Table S2. In detail, 1,466 DEGs were identified in SA, with 484 upregulated and 982 downregulated compared to CON. In the SP vs CON comparison, 2,009 DEGs were selected, with 708 upregulated and 1,301 downregulated. Finally, in the SB vs CON comparison, 696 DEGs were identified, with 368 upregulated and 328 downregulated. A Venn plot demonstrated the number of single- or co-expressed DEGs (Fig. 5C; Table S3). Among all comparisons, 136 DEGs, including *FAM64A*, *IGFN1*, *IL17B*, *MYOM2*, and *TNFSF11*, were screened, and their expression levels among the four groups are shown in Fig. 5D. These genes can be suggested as marker genes for hindgut epithelial responses to VFAs.

### Functional enrichment analysis of DEGs

**Fig 6:**
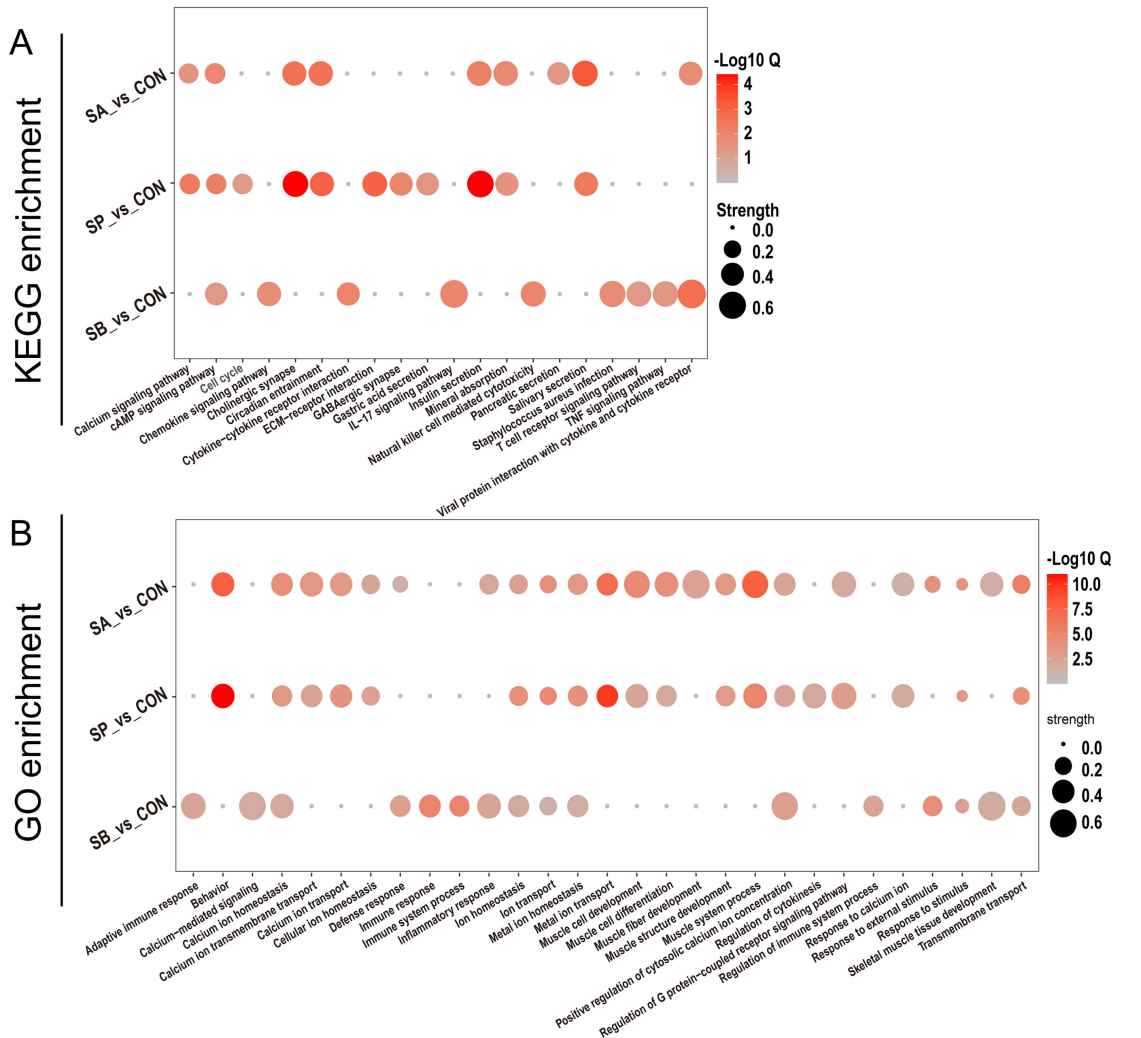
The KEGG and GO functional enrichment analyses of DEGs. (**A**) The functional enrichment analysis of DEGs using the KEGG database; (**B**) the functional enrichment analysis of DEGs using the GO database.

The up- and downregulated DEGs underwent functional enrichment analysis using KEGG and GO databases. Results revealed that the administration of each VFA contributed to different epithelial responses. For KEGG enrichment (Fig. 6A), most DEGs between SA and CON were enriched in pathways such as salivary secretion, insulin secretion, mineral absorption, calcium signaling, and pancreatic secretion. Between SP and CON, most DEGs were enriched in pathways including salivary secretion, circadian entrainment, cholinergic synapse, insulin secretion, mineral absorption, and gastric acid secretion. Finally, DEGs between SB and CON were enriched in pathways related to viral protein interaction with cytokine and cytokine receptor, cytokine-cytokine receptor interaction, interleukin-17 (IL-17) signaling pathway, TNF signaling pathway, and T cell receptor signaling pathway.

The GO enrichment results also indicated varying degrees of biological process impact from the VFA administration (Fig. 6B). For instance, most DEGs were enriched in behavior, muscle development and differentiation, and metal ion (such as calcium ion) homeostasis and transport-related pathways. Several immune response and inflammatory pathways were highly enriched between SB and CON.

### The protein concentration of inflammatory cytokines and tight junctions in hindgut epithelium

The protein concentration of inflammatory cytokines and tight junctions in hindgut epithelium was measured using ELISA methods (Table 5). For inflammatory cytokines in colonic epithelium, data revealed that administration of three VFAs had no effect on IL-1β (*P* = 0.196), IL-6 (*P* = 0.204), and TNF-α (*P* = 0.238), while SA and SP increased the concentration of IL-10 compared with CON and SB (*P* = 0.001). Regarding tight junctions in colonic epithelium, the concentration of Claudin1 in SB was higher than that in SA and SP (*P* = 0.01), while the concentration of ZO-1 in SA was higher than that in CON (*P* = 0.034). The differences in Occludin were not significant among the four groups (*P* = 0.934). Similar results were found in the cecal epithelium. SA and SP significantly increased the concentration of IL-10 compared with CON and SB (*P* = 0.001). Administration of three VFAs and saline did not affect the concentrations of IL-1β (*P* = 0.16), IL-6 (*P* = 0.135), and TNF-α (*P* = 0.277). For tight junctions in cecal epithelium, SA increased the concentration of Claudin1 (*P* = 0.01) compared with the other three groups, while the differences in Occludin (*P* = 0.766) and ZO-1 (*P* = 0.166) were not significant among the four groups.

**TABLE 5:** The concentrations of inflammatory cytokines and tight junctions in colonic and cecal epithelial tissues^*a*^

Items	CON	SA	SP	SB	SEM	*P*-value
Colonic epithelial tissue						
IL-1β (pg/mg protein)	14.88	15.47	15.54	16.90	0.342	0.196
IL-6 (pg/mg protein)	33.03	32.07	29.24	30.14	0.699	0.204
IL-10 (pg/mg protein)	40.91c	48.60a	45.74ab	44.07bc	0.785	0.001
TNF-α (pg/mg protein)	119.53	118.28	122.29	107.99	2.614	0.238
Claudin1 (pg/mg protein)	11.21ab	10.39bc	9.97c	11.58a	0.204	0.010
Occludin (ng/mg protein)	1.19	1.14	1.18	1.18	0.030	0.934
ZO-1 (ng/mg protein)	115.80b	134.55a	126.13ab	124.48ab	2.359	0.034
Cecal epithelial tissue						
IL-1β (pg/mg protein)	27.30	29.14	26.34	26.93	0.462	0.160
IL-6 (pg/mg protein)	57.47	56.86	59.62	53.84	0.884	0.135
IL-10 (pg/mg protein)	19.34b	22.45a	22.11a	18.61b	0.427	0.001
TNF-α (pg/mg protein)	210.07	223.90	221.44	214.76	2.746	0.277
Claudin1 (pg/mg protein)	21.16b	22.54a	20.20bc	19.90c	0.281	0.001
Occludin (ng/mg protein)	2.21	2.18	2.18	2.27	0.030	0.766
ZO-1 (ng/mg protein)	213.42	217.07	223.05	199.51	3.847	0.166

^*a*^ Values with different letters within a row mean statistically significant (*P* < 0.05 or *P* < 0.01).

### The interactions between candidate bacteria and homeostasis status, metabolome, and transcriptome sequencing data

**Fig 7:**
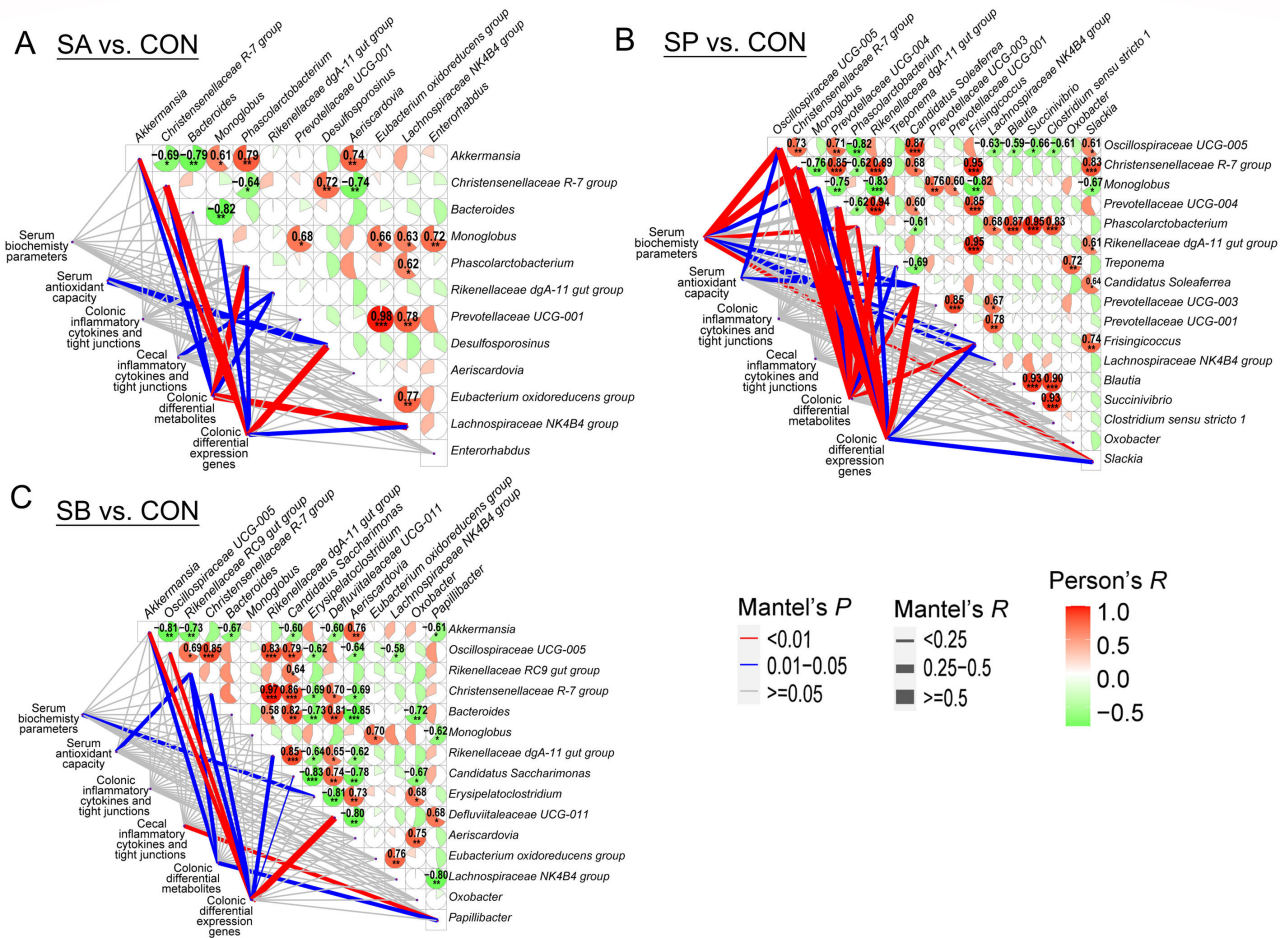
The interactions between candidate bacteria and homeostasis status, metabolome, and transcriptome sequencing data. (**A**) Correlation analysis between SA and CON; (**B**) correlation analysis between SP and CON; (**C**) correlation analysis between SB and CON; the correlation analysis of candidate bacteria was calculated using Pearson’s correlation coefficient. *P < 0.05; **P < 0.01; and ***P < 0.001. The correlation analysis between candidate bacteria and homeostasis status, metabolome, and transcriptome sequencing data was performed using Mantel’s test. Blue line indicates Mantel’s *P* < 0.05, red line indicates Mantel’s *P* < 0.01, and the gray line indicates Mantel’s *P* > 0.05.

The interactions between candidate bacteria and hindgut homeostasis, as well as multi-omics data in response to different VFA administrations, were studied using Pearson’s correlation and Mantel’s test (Fig. 7). Most of these gut bacteria were correlated with each other, as well as differential metabolites and DEGs in gut epithelium; however, these bacteria were rarely correlated with inflammatory cytokines and tight junctions. For example, between SA and CON (Fig. 7A), *Christensenellaceae* R-7 group was positively correlated with *Desulfosporosinus* (Pearson’s *P* < 0.01); meanwhile, the *Christensenellaceae* R-7 group was correlated with differential metabolites (Mantel’s *P* < 0.05) and DEGs (Mantel’s *P* < 0.01), while *Desulfosporosinus* was correlated with serum antioxidant capacity parameters (Mantel’s *P* < 0.05) and DEGs (Mantel’s *P* < 0.01). Between SP and CON (Fig. 7B), *Prevotellaceae* UCG-004 was positively correlated with *Frisingicoccus* (Pearson’s *P* < 0.001), and both of these two bacteria were correlated with differential metabolites (Mantel’s *P* < 0.01) and DEGs (Mantel’s *P* < 0.05 or Mantel’s *P* < 0.01). Between SB and CON (Fig. 7C), *Akkermansia* was negatively correlated with *Oscillospiraceae* UCG-005 (Pearson’s *P* < 0.01), and both of these two bacteria were correlated with DEGs (Mantel’s *P* < 0.01).

## DISCUSSION

In most studies reported in the literature, supplementation with VFAs has shown several advantages for ruminants. For example, SA has been demonstrated to increase milk yield (46) and promote milk fat synthesis (47, 48), while SP can improve nitrogen utilization, glucose metabolism, and protect the blood-milk barrier integrity (49–51). SB has also been reported to promote rumen papillae development and reduce inflammation and autophagy in the rumen epithelium (24, 52, 53). However, limited data are available on the role of VFAs in hindgut bacteria, metabolism, and epithelial homeostasis in dairy goats. In our study, we systematically evaluated the effects of VFAs on hindgut bacterial composition and metabolism, epithelial homeostasis parameters, and transcriptome profiling in dairy goats.

It is well-known that the majority of VFAs produced in the rumen are absorbed through the rumen wall, but a portion does pass to the lower digestive tract (54). Our study demonstrated that oral administration of VFAs into the rumen also had a significant impact on the concentration of VFAs in the hindgut. In detail, data showed that SA significantly increased the concentration of total VFAs, acetate, and other VFAs (valerate, isobutyrate, and isovalerate) in the cecal contents compared with the other groups. SB also significantly increased the concentration of butyrate compared with CON and SP. These results indicated that despite the primary absorption of VFAs through the rumen, sufficient quantities of administered VFAs reached the cecum, thereby influencing the microbial composition and metabolic activities. This is supported by the dynamics of VFA absorption and passage rates from rumen to cecum, and the variations in bacterial fermentation patterns and metabolic utilization of VFAs of the rumen from our previous study (27). Furthermore, we reported that VFA administration decreased the expression levels of VFA absorption genes, such as *MCT1* and *MCT4* in the rumen epithelium. This may lead to changes in the systemic absorption and metabolism of VFAs, further affecting the production of VFAs in the cecum (27). Moreover, the difference between the rumen and the hindgut of ruminants lies in the lack of factors such as saliva and protozoa, resulting in weak buffering ability, and the VFAs entering the hindgut cannot be absorbed by the intestinal epithelium in time, resulting in changes in VFA concentration (12, 54).

The 16S rRNA sequencing data demonstrated that VFA administration significantly impacted the succession of cecal bacteria. SA increased the relative abundance of *Bacteroides, Christensenellaceae* R-7 group, *Rikenellaceae* dgA-11 gut group, and *Desulfosporosinus*; SP increased the relative abundance of *Oscillospiraceae* UCG-005*, Prevotellaceae* UCG-004, *Christensenellaceae* R-7 group, *Slackia*, *Rikenellaceae* dgA-11 gut group, *Candidatus* Soleaferrea, and *Frisingicoccus;* and SB increased the relative abundance of *Oscillospiraceae* UCG-005*, Rikenellaceae* RC9 gut group, *Rikenellaceae* dgA-11 gut group, *Christensenellaceae* R-7 group, *Bacteroides*, *Candidatus* Saccharimonas, *Defluviitaleaceae* UCG-011, and *Papillibacter*. Previous studies have linked these bacteria, such as *Bacteroides*, *Rikenellaceae* dgA-11 gut group, *Christensenellaceae*, *Papillibacter*, and *Rikenellaceae* RC9 gut group*,* with the production of VFAs (23, 55–57). Therefore, our data indicated that VFAs could significantly affect the abundances of VFA-producing bacteria, thereby altering microbial fermentation and metabolic patterns. Interestingly, administration with three VFAs increased the relative abundance of *Christensenellaceae* R-7 group in the cecum, a gut bacterium that is reported to be more abundant in healthy people than in people with inflammatory bowel disease, suggesting that VFA administration may benefit gut health in goats (58). The SA-induced reduction in Chao1 index was consistent with a significant enrichment of specialized taxa, including *Bacteroides*, *Christensenellaceae* R-7 group, and *Rikenellaceae* dgA-11 gut group, accompanied by a decrease of mucin-degrading bacteria *Akkermansia.* Similar alterations in microbial abundances were also observed in the SB administration group, along with a downward trend in the Chao1 index in SB. These taxonomic shifts suggested that high acetate availability favors fast-growing, acetate-utilizing bacteria (such as *Bacteroides*), which may outcompete rare species through niche specialization, thereby reducing overall richness while promoting functional dominance (59, 60).

Our findings indicated that VFA administration had a limited effect on systemic lipid metabolism in goats, which was supported by insignificant differences in serum TG and CHOL concentrations among the four groups. This result could reflect the efficient energy utilization strategy in ruminants. The administered VFAs may be directly absorbed by the rumen and intestinal epithelium and further oxidized for energy supplementation rather than being used for lipid synthesis, thereby maintaining the systemic lipid homeostasis (61). Furthermore, data revealed that VFA administration could enhance gut homeostasis by modulating inflammatory responses and tight junctions. For instance, SB decreased the MDA level in serum, which could alleviate systemic oxidative stress (62). Additionally, SA and SP increased the level of IL-10 in both colonic and cecal epithelium, indicating their anti-inflammatory effects in gut epithelium, which further improve the resistance of the intestinal epithelium to pathogen invasion and enhance the barrier functions (62, 63). The ELISA results also suggested that different VFA administrations increased the concentration of tight junctions. SB increased the protein concentration of Claudin1 in colonic epithelium, while SA increased the protein concentration of ZO-1 in colonic epithelium and Claudin1 in cecal epithelium. It has been previously reported that the Th1, Th2, and Th17 cytokines play an important role in maintaining gut homeostasis (64, 65). Furthermore, VFAs, particularly butyrate, exert their protective roles in gut inflammation through the inhibition of histone deacetylases (HDACs, such as HDAC3) in gut epithelium (66). VFAs can suppress the differentiation of Th1 and Th17 cells to reduce the production of pro-inflammatory cytokines (such as IFN-γ, TNF, and IL-17), while promoting Th2-mediated secretion of the anti-inflammatory cytokines (such as IL-10) (67, 68). Our findings aligned with the observed dual immunomodulatory effects of VFAs. The reduction level of MDA and the increased concentrations of IL-10 and ZO-1/Claudin1 tight junctions demonstrated that the VFAs had positive roles in alleviating oxidative stress, mitigating inflammatory responses, and preserving the integrity of gut barrier functions in goats. These findings could provide guidance for the prevention and management of subacute acidosis induced by high-concentrate diet in ruminants (69).

VFA administration significantly reshaped hindgut metabolic profiles, with purine, tryptophan, phenylalanine, and tyrosine metabolism being the most responsive pathways. Purine metabolites regulate energy homeostasis and signaling transduction, while tryptophan derivatives (kynurenic acid and 5-hydroxyindoleacetic acid) modulate immunity and anti-inflammation functions (70–73). Notably, SA treatment reduced cecal microbial richness (Chao1), driven by the dominance of acetate-utilizing taxa (*Bacteroides* and *Christensenellaceae* R-7 group) and the reduction of *Akkermansia*. This taxonomic shift correlated with decreased purine (3′-AMP, AMP), steroid (androstenedione), and amino acid (L-proline) metabolites, indicating reduced metabolic redundancy. However, SA upregulated anti-inflammatory and antioxidant metabolites, such as 5-hydroxyindoleacetic acid, urocanic acid, and 3,4-dihydroxymandelic acid (ROS scavenger), suggesting compensatory mechanisms to maintain gut homeostasis (74, 75). These findings demonstrated that SA-induced microbial simplification prioritizes niche efficiency (antioxidant capacity, anti-inflammation, and barrier function) over metabolic versatility, offering a targeted strategy to optimize gut health by acetate-driven microbiota remodeling.

The functional enrichment analysis of transcriptome sequencing data of colonic epithelium suggested that VFA administration had varying effects on nutrient absorption and transport, development, homeostasis, and immunity of colonic epithelium. SA and SP affected endocrine and digestive functions, metal ion (particularly calcium ion) homeostasis and transport, and muscle development and differentiation in colonic epithelium. Several immune response and inflammation-related pathways were especially enriched in SB. Intracellular calcium ions play a crucial role in maintaining biopotential, nerve conduction, and energy absorption in intestinal epithelial cells of ruminants (76). Previous studies have shown that VFAs can activate intracellular calcium ion signaling in rats (77). Our study reported that VFAs can also regulate calcium ion signaling pathways in the hindgut of dairy goats. Additionally, muscle development and differentiation pathways were enriched in SA compared with CON, indicating that VFAs, such as acetate, can regulate energy metabolism in the colon (78). Finally, apart from SA and SP, our results explained that SB had its unique role in regulating intestinal epithelial homeostasis and immunity, similar to studies in the rumen (79–81).

### Conclusions

This study highlighted the significant roles of VFAs on hindgut bacterial metabolism and homeostasis and provided novel insights into the metabolic and physiological roles beyond the rumen. VFA administration positively affects hindgut epithelial health and barrier function. Specifically, SB reduced serum MDA levels, indicating a decrease in oxidative stress, whereas SA and SP increased IL-10 concentration, reflecting an enhanced anti-inflammatory response. VFA administration also contributed to gut barrier integrity by increasing the expression of tight junctions. Data also revealed that VFAs modulated microbial communities, and the changes in microbial composition were accompanied by significant shifts in metabolic pathways. Transcriptomic analysis further demonstrated that SA and SP influenced varying aspects of colonic epithelial function, including endocrine activity, digestive processes, metal ion homeostasis, and muscle development. Additionally, immune response pathways were enriched in SB. These findings provided a comprehensive understanding of the nutritional regulatory effects of VFAs and suggested potential strategies for improving gut health and overall well-being in ruminants through dietary interventions.

## Data Availability

The RNA-seq data have been deposited in the NCBI Gene Expression Omnibus (GEO) database under accession code GSE232035 (https://www.ncbi.nlm.nih.gov/geo/query/acc.cgi?acc=GSE232035), while the 16S rRNA sequencing data have been deposited in the GEO database under accession code GSE232036 (https://www.ncbi.nlm.nih.gov/geo/query/acc.cgi?acc=GSE232036). The LC-MS metabolome data were deposited in the OMIX database of the China National Center for Bioinformation under accession number OMIX006440 (https://ngdc.cncb.ac.cn/omix/release/OMIX006440). Additional data related to this paper may be requested from the authors.

## SUPPLEMENTAL MATERIAL

Table S1 msystems.00116-25-s0001.xlsx Significantly different metabolites of three comparisons in cecal contents.

Table S2 msystems.00116-25-s0002.xlsx Expression levels of DEGs in colonic epithelium.

Table S3 msystems.00116-25-s0003.xlsx DEGs expressed separately and co-expressed in colonic epithelium.

ASM does not own the copyrights to Supplemental Material that may be linked to, or accessed through, an article. The authors have granted ASM a non-exclusive, world-wide license to publish the Supplemental Material files. Please contact the corresponding author directly for reuse.

## References

[1] Mottet A, Teillard F, Boettcher P, De’ Besi G, Besbes B. 2018. Review: domestic herbivores and food security: current contribution, trends and challenges for a sustainable development. Animal 12:s188–s198. doi:10.1017/S1751731118002215 30215340

[2] Ranadheera CS, Evans CA, Baines SK, Balthazar CF, Cruz AG, Esmerino EA, Freitas MQ, Pimentel TC, Wittwer AE, Naumovski N, Graça JS, Sant’Ana AS, Ajlouni S, Vasiljevic T. 2019. Probiotics in goat milk products: delivery capacity and ability to improve sensory attributes. Compr Rev Food Sci Food Saf 18:867–882. doi:10.1111/1541-4337.12447 33337004

[3] Ranadheera CS, Naumovski N, Ajlouni S. 2018. Non-bovine milk products as emerging probiotic carriers: recent developments and innovations. Curr Opin Food Sci 22:109–114. doi:10.1016/j.cofs.2018.02.010

[4] Nayik GA, Jagdale YD, Gaikwad SA, Devkatte AN, Dar AH, Dezmirean DS, Bobis O, Ranjha M, Ansari MJ, Hemeg HA, Alotaibi SS. 2021. Recent insights into processing approaches and potential health benefits of goat milk and its products: a review. Front Nutr 8:789117. doi:10.3389/fnut.2021.789117 34938763 PMC8685332

[5] Eisler MC, Lee MRF, Tarlton JF, Martin GB, Beddington J, Dungait JAJ, Greathead H, Liu J, Mathew S, Miller H, Misselbrook T, Murray P, Vinod VK, Van Saun R, Winter M. 2014. Agriculture: dteps to sustainable livestock. Nature 507:32–34. doi:10.1038/507032a 24605375

[6] Dill-McFarland KA, Weimer PJ, Breaker JD, Suen G. 2019. Diet influences early microbiota development in dairy calves without long-term impacts on milk production. Appl Environ Microbiol 85:e02141-18. doi:10.1128/AEM.02141-18 30367001 PMC6328763

[7] Chen L, Qiu Q, Jiang Y, Wang K, Lin Z, Li Z, Bibi F, Yang Y, Wang J, Nie W, et al.. 2019. Large-scale ruminant genome sequencing provides insights into their evolution and distinct traits. Science 364:eaav6202. doi:10.1126/science.aav6202 31221828

[8] Lin L, Lai Z, Zhang J, Zhu W, Mao S. 2023. The gastrointestinal microbiome in dairy cattle is constrained by the deterministic driver of the region and the modified effect of diet. Microbiome 11:10. doi:10.1186/s40168-022-01453-2 36670455 PMC9863278

[9] Mizrahi I, Wallace RJ, Moraïs S. 2021. The rumen microbiome: balancing food security and environmental impacts. Nat Rev Microbiol 19:553–566. doi:10.1038/s41579-021-00543-6 33981031

[10] Moraïs S, Mizrahi I. 2019. The road not taken: the rumen microbiome, functional groups, and community states. Trends Microbiol 27:538–549. doi:10.1016/j.tim.2018.12.011 30679075

[11] van Gastelen S, Dijkstra J, Alferink SJJ, Binnendijk G, Nichols K, Zandstra T, Bannink A. 2021. Abomasal infusion of corn starch and β-hydroxybutyrate in early-lactation Holstein-Friesian dairy cows to induce hindgut and metabolic acidosis. J Dairy Sci 104:12520–12539. doi:10.3168/jds.2021-20323 34482977

[12] Xie F, Xu L, Wang Y, Mao S. 2021. Metagenomic sequencing reveals that high-grain feeding alters the composition and metabolism of cecal microbiota and induces cecal mucosal injury in sheep. mSystems 6:e0091521. doi:10.1128/msystems.00915-21 34609166 PMC8547435

[13] Wang MY, Li Y, Gao M, Song LW, Xu M, Zhao XL, Jia Y, Zhao M, Sun YY, Hu HL. 2021. Effects of subacute ruminal acidosis on colon epithelial morphological structure, permeability, and expression of key tight junction proteins in dairy goats. J Dairy Sci 104:4260–4270. doi:10.3168/jds.2020-18738 33485680

[14] Xie F, Jin W, Si H, Yuan Y, Tao Y, Liu J, Wang X, Yang C, Li Q, Yan X, Lin L, Jiang Q, Zhang L, Guo C, Greening C, Heller R, Guan LL, Pope PB, Tan Z, Zhu W, Wang M, Qiu Q, Li Z, Mao S. 2021. An integrated gene catalog and over 10,000 metagenome-assembled genomes from the gastrointestinal microbiome of ruminants. Microbiome 9:137. doi:10.1186/s40168-021-01078-x 34118976 PMC8199421

[15] Lyte M, Villageliú DN, Crooker BA, Brown DR. 2018. Symposium review: microbial endocrinology—Why the integration of microbes, epithelial cells, and neurochemical signals in the digestive tract matters to ruminant health. J Dairy Sci 101:5619–5628. doi:10.3168/jds.2017-13589 29550113

[16] Gu F, Zhu S, Hou J, Tang Y, Liu JX, Xu Q, Sun HZ. 2023. The hindgut microbiome contributes to host oxidative stress in postpartum dairy cows by affecting glutathione synthesis process. Microbiome 11:87. doi:10.1186/s40168-023-01535-9 37087457 PMC10122372

[17] Zhang K, Xu Y, Yang Y, Guo M, Zhang T, Zong B, Huang S, Suo L, Ma B, Wang X, Wu Y, Brugger D, Chen Y. 2022. Gut microbiota-derived metabolites contribute negatively to hindgut barrier function development at the early weaning goat model. Anim Nutr 10:111–123. doi:10.1016/j.aninu.2022.04.004 35663372 PMC9136126

[18] Abeyta MA, Horst EA, Goetz BM, Rodriguez-Jimenez S, Mayorga EJ, Al-Qaisi M, Baumgard LH. 2023. Effects of hindgut acidosis on inflammation, metabolism, and productivity in lactating dairy cows fed a high-fiber diet. J Dairy Sci 106:2879–2889. doi:10.3168/jds.2022-22680 36823004

[19] Na SW, Guan LL. 2022. Understanding the role of rumen epithelial host-microbe interactions in cattle feed efficiency. Anim Nutr 10:41–53. doi:10.1016/j.aninu.2022.04.002 35647325 PMC9117530

[20] McCurdy DE, Wilkins KR, Hiltz RL, Moreland S, Klanderman K, Laarman AH. 2019. Effects of supplemental butyrate and weaning on rumen fermentation in Holstein calves. J Dairy Sci 102:8874–8882. doi:10.3168/jds.2019-16652 31351719

[21] Górka P, Kowalski ZM, Pietrzak P, Kotunia A, Jagusiak W, Holst JJ, Guilloteau P, Zabielski R. 2011. Effect of method of delivery of sodium butyrate on rumen development in newborn calves. J Dairy Sci 94:5578–5588. doi:10.3168/jds.2011-4166 22032381

[22] Zhong H, Yu W, Wang M, Lin B, Sun X, Zheng N, Wang J, Zhao S. 2023. Sodium butyrate promotes gastrointestinal development of preweaning bull calves via inhibiting inflammation, balancing nutrient metabolism, and optimizing microbial community functions. Anim Nutr 14:88–100. doi:10.1016/j.aninu.2023.04.004 37388163 PMC10300058

[23] Wu W, Lu H, Cheng J, Geng Z, Mao S, Xue Y. 2023. Undernutrition disrupts cecal microbiota and epithelium interactions, epithelial metabolism, and immune responses in a pregnant sheep model. Microbiol Spectr 11:e05320-22. doi:10.1128/spectrum.05320-22 36976022 PMC10100782

[24] Liu L, Sun D, Mao S, Zhu W, Liu J. 2019. Infusion of sodium butyrate promotes rumen papillae growth and enhances expression of genes related to rumen epithelial VFA uptake and metabolism in neonatal twin lambs. J Anim Sci 97:909–921. doi:10.1093/jas/sky459 30535158 PMC6377441

[25] Gualdrón-Duarte LB, Allen MS. 2018. Effects of acetic acid or sodium acetate infused into the rumen or abomasum on feeding behavior and metabolic response of cows in the postpartum period. J Dairy Sci 101:2016–2026. doi:10.3168/jds.2017-13609 29398027

[26] Bedford A, Beckett L, Hardin K, Dias NW, Davis T, Mercadante VRG, Ealy AD, White RR. 2018. Propionate affects insulin signaling and progesterone profiles in dairy heifers. Sci Rep 8:17629. doi:10.1038/s41598-018-35977-1 30514961 PMC6279792

[27] Zhen Y, Xi Z, Nasr SM, He F, Han M, Yin J, Ge L, Chen Y, Wang Y, Wei W, Zhang Y, Wang M. 2023. Multi-omics reveals the impact of exogenous short-chain fatty acid infusion on rumen homeostasis: insights into crosstalk between the microbiome and the epithelium in a goat model. Microbiol Spectr 11:e05343-22. doi:10.1128/spectrum.05343-22 37439665 PMC10433986

[28] Van Soest PJ, Robertson JB, Lewis BA. 1991. Methods for dietary fiber, neutral detergent fiber, and nonstarch polysaccharides in relation to animal nutrition. J Dairy Sci 74:3583–3597. doi:10.3168/jds.S0022-0302(91)78551-2 1660498

[29] Logue JB, Stedmon CA, Kellerman AM, Nielsen NJ, Andersson AF, Laudon H, Lindström ES, Kritzberg ES. 2016. Experimental insights into the importance of aquatic bacterial community composition to the degradation of dissolved organic matter. ISME J 10:533–545. doi:10.1038/ismej.2015.131 26296065 PMC4817675

[30] Magoč T, Salzberg SL. 2011. FLASH: fast length adjustment of short reads to improve genome assemblies. Bioinformatics 27:2957–2963. doi:10.1093/bioinformatics/btr507 21903629 PMC3198573

[31] Rognes T, Flouri T, Nichols B, Quince C, Mahé F. 2016. VSEARCH: a versatile open source tool for metagenomics. PeerJ 4:e2584. doi:10.7717/peerj.2584 27781170 PMC5075697

[32] Callahan BJ, McMurdie PJ, Rosen MJ, Han AW, Johnson AJA, Holmes SP. 2016. DADA2: high-resolution sample inference from Illumina amplicon data. Nat Methods 13:581–583. doi:10.1038/nmeth.3869 27214047 PMC4927377

[33] Bolyen E, Rideout JR, Dillon MR, Bokulich NA, Abnet CC, Al-Ghalith GA, Alexander H, Alm EJ, Arumugam M, Asnicar F, et al.. 2019. Reproducible, interactive, scalable and extensible microbiome data science using QIIME 2. Nat Biotechnol 37:852–857. doi:10.1038/s41587-019-0209-9 31341288 PMC7015180

[34] Bokulich NA, Subramanian S, Faith JJ, Gevers D, Gordon JI, Knight R, Mills DA, Caporaso JG. 2013. Quality-filtering vastly improves diversity estimates from Illumina amplicon sequencing. Nat Methods 10:57–59. doi:10.1038/nmeth.2276 23202435 PMC3531572

[35] Segata N, Izard J, Waldron L, Gevers D, Miropolsky L, Garrett WS, Huttenhower C. 2011. Metagenomic biomarker discovery and explanation. Genome Biol 12:R60. doi:10.1186/gb-2011-12-6-r60 21702898 PMC3218848

[36] Zelena E, Dunn WB, Broadhurst D, Francis-McIntyre S, Carroll KM, Begley P, O’Hagan S, Knowles JD, Halsall A, Wilson ID, Kell DB, HUSERMET Consortium. 2009. Development of a robust and repeatable UPLC-MS method for the long-term metabolomic study of human serum. Anal Chem 81:1357–1364. doi:10.1021/ac8019366 19170513

[37] Want EJ, Masson P, Michopoulos F, Wilson ID, Theodoridis G, Plumb RS, Shockcor J, Loftus N, Holmes E, Nicholson JK. 2013. Global metabolic profiling of animal and human tissues via UPLC-MS. Nat Protoc 8:17–32. doi:10.1038/nprot.2012.135 23222455

[38] Smith CA, Want EJ, O’Maille G, Abagyan R, Siuzdak G. 2006. XCMS: processing mass spectrometry data for metabolite profiling using nonlinear peak alignment, matching, and identification. Anal Chem 78:779–787. doi:10.1021/ac051437y 16448051

[39] Navarro-Reig M, Jaumot J, García-Reiriz A, Tauler R. 2015. Evaluation of changes induced in rice metabolome by Cd and Cu exposure using LC-MS with XCMS and MCR-ALS data analysis strategies. Anal Bioanal Chem 407:8835–8847. doi:10.1007/s00216-015-9042-2 26403240

[40] Tsugawa H, Tsujimoto Y, Arita M, Bamba T, Fukusaki E. 2011. GC/MS based metabolomics: development of a data mining system for metabolite identification by using soft independent modeling of class analogy (SIMCA). BMC Bioinform 12:131. doi:10.1186/1471-2105-12-131 PMC3102042 21542920

[41] Xia J, Wishart DS. 2011. Web-based inference of biological patterns, functions and pathways from metabolomic data using MetaboAnalyst. Nat Protoc 6:743–760. doi:10.1038/nprot.2011.319 21637195

[42] Chen S, Zhou Y, Chen Y, Gu J. 2018. fastp: an ultra-fast all-in-one FASTQ preprocessor. Bioinformatics 34:i884–i890. doi:10.1093/bioinformatics/bty560 30423086 PMC6129281

[43] Pertea M, Kim D, Pertea GM, Leek JT, Salzberg SL. 2016. Transcript-level expression analysis of RNA-seq experiments with HISAT, StringTie and Ballgown. Nat Protoc 11:1650–1667. doi:10.1038/nprot.2016.095 27560171 PMC5032908

[44] Bickhart DM, Rosen BD, Koren S, Sayre BL, Hastie AR, Chan S, Lee J, Lam ET, Liachko I, Sullivan ST, et al.. 2017. Single-molecule sequencing and chromatin conformation capture enable de novo reference assembly of the domestic goat genome. Nat Genet 49:643–650. doi:10.1038/ng.3802 28263316 PMC5909822

[45] Li H, Handsaker B, Wysoker A, Fennell T, Ruan J, Homer N, Marth G, Abecasis G, Durbin R, 1000 Genome Project Data Processing Subgroup. 2009. The sequence Alignment/Map format and SAMtools. Bioinformatics 25:2078–2079. doi:10.1093/bioinformatics/btp352 19505943 PMC2723002

[46] Matamoros C, Hao F, Tian Y, Patterson AD, Harvatine KJ. 2022. Interaction of sodium acetate supplementation and dietary fiber level on feeding behavior, digestibility, milk synthesis, and plasma metabolites. J Dairy Sci 105:8824–8838. doi:10.3168/jds.2022-21911 36175230

[47] Luo C, Li N, Wang Q, Li C. 2023. Sodium acetate promotes fat synthesis by suppressing TATA element modulatory factor 1 in bovine mammary epithelial cells. Anim Nutr 13:126–136. doi:10.1016/j.aninu.2023.01.002 37123620 PMC10130354

[48] Matamoros C, Salfer IJ, Bartell PA, Harvatine KJ. 2022. Effect of the timing of sodium acetate infusion on the daily rhythms of milk synthesis and plasma metabolites and hormones in Holstein cows. J Dairy Sci 105:7432–7445. doi:10.3168/jds.2022-21912 35931478

[49] Agarwal U, Hu Q, Bequette BJ. 2015. Propionate supplementation improves nitrogen use by reducing urea flux in sheep. J Anim Sci 93:4883–4890. doi:10.2527/jas.2015-9226 26523581

[50] Kennedy KM, Donkin SS, Allen MS. 2020. Effects of propionate concentration on short-term metabolism in liver explants from dairy cows in the postpartum period. J Dairy Sci 103:11449–11460. doi:10.3168/jds.2020-18914 33222857

[51] Ali I, Yang M, Wang Y, Yang C, Shafiq M, Wang G, Li L. 2021. Sodium propionate protect the blood-milk barrier integrity, relieve lipopolysaccharide-induced inflammatory injury and cells apoptosis. Life Sci 270:119138. doi:10.1016/j.lfs.2021.119138 33524422

[52] Li Y, Liu J, Cui Y, Cao Y, Xu P, Kan X, Guo W, Fu S. 2022. Sodium butyrate attenuates bovine mammary epithelial cell injury by inhibiting the formation of neutrophil extracellular traps. Int Immunopharmacol 110:109009. doi:10.1016/j.intimp.2022.109009 35816944

[53] Yang T, Datsomor O, Jiang M, Ma X, Zhao G, Zhan K. 2022. Protective roles of sodium butyrate in lipopolysaccharide-induced bovine ruminal epithelial cells by activating g protein-coupled receptors 41. Front Nutr 9:842634. doi:10.3389/fnut.2022.842634 35600833 PMC9121101

[54] Gressley TF, Hall MB, Armentano LE. 2011. Ruminant Nutrition Symposium: productivity, digestion, and health responses to hindgut acidosis in ruminants. J Anim Sci 89:1120–1130. doi:10.2527/jas.2010-3460 21415422

[55] Zhao Y, Xie B, Gao J, Zhao G. 2020. Dietary supplementation with sodium sulfate improves rumen fermentation, fiber digestibility, and the plasma metabolome through modulation of rumen bacterial communities in steers. Appl Environ Microbiol 86:e01412-20. doi:10.1128/AEM.01412-20 32859601 PMC7642074

[56] Ren T, Xu M, Zhou S, Ren J, Li B, Jiang P, Li H, Wu W, Chen C, Fan M, Jiao L. 2023. Structural characteristics of mixed pectin from ginseng berry and its anti-obesity effects by regulating the intestinal flora. Int J Biol Macromol 242:124687. doi:10.1016/j.ijbiomac.2023.124687 37146855

[57] Guo H, Li B, Gao M, Li Q, Gao Y, Dong N, Liu G, Wang Z, Gao W, Chen Y, Yang Y. 2022. Dietary nutritional level affects intestinal microbiota and health of goats. Microorganisms 10:2322. doi:10.3390/microorganisms10122322 36557575 PMC9781347

[58] Goodrich JK, Waters JL, Poole AC, Sutter JL, Koren O, Blekhman R, Beaumont M, Van Treuren W, Knight R, Bell JT, Spector TD, Clark AG, Ley RE. 2014. Human genetics shape the gut microbiome. Cell 159:789–799. doi:10.1016/j.cell.2014.09.053 25417156 PMC4255478

[59] Makki K, Deehan EC, Walter J, Bäckhed F. 2018. The impact of dietary fiber on gut microbiota in host health and disease. Cell Host Microbe 23:705–715. doi:10.1016/j.chom.2018.05.012 29902436

[60] Culp EJ, Goodman AL. 2023. Cross-feeding in the gut microbiome: ecology and mechanisms. Cell Host Microbe 31:485–499. doi:10.1016/j.chom.2023.03.016 37054671 PMC10125260

[61] Newbold CJ, Ramos-Morales E. 2020. Review: ruminal microbiome and microbial metabolome: effects of diet and ruminant host. Animal 14:s78–s86. doi:10.1017/S1751731119003252 32024572

[62] Zhang C, Hou T, Wang J, Yu Q, Zhang Y, Sun Y. 2023. Clostridium butyricum alleviates LPS-induced acute immune stress in goats by regulating bacterial communities and blood metabolites. Front Immunol 14:1099186. doi:10.3389/fimmu.2023.1099186 36756118 PMC9899838

[63] Kashiwagi I, Morita R, Schichita T, Komai K, Saeki K, Matsumoto M, Takeda K, Nomura M, Hayashi A, Kanai T, Yoshimura A. 2015. Smad2 and Smad3 inversely regulate TGF-β autoinduction in Clostridium butyricum-activated dendritic cells. Immunity 43:65–79. doi:10.1016/j.immuni.2015.06.010 26141582

[64] Pandey SP, Yan J, Turner JR, Abraham C. 2019. Reducing IRF5 expression attenuates colitis in mice, but impairs the clearance of intestinal pathogens. Mucosal Immunol 12:874–887. doi:10.1038/s41385-019-0165-1 31053739 PMC6688861

[65] Yan J, Pandey SP, Barnes BJ, Turner JR, Abraham C. 2020. T cell-intrinsic IRF5 regulates T cell signaling, migration, and differentiation and promotes intestinal inflammation. Cell Rep 31:107820. doi:10.1016/j.celrep.2020.107820 32610123 PMC7409536

[66] Mann ER, Lam YK, Uhlig HH. 2024. Short-chain fatty acids: linking diet, the microbiome and immunity. Nat Rev Immunol 24:577–595. doi:10.1038/s41577-024-01014-8 38565643

[67] Park J, Kim M, Kang SG, Jannasch AH, Cooper B, Patterson J, Kim CH. 2015. Short-chain fatty acids induce both effector and regulatory T cells by suppression of histone deacetylases and regulation of the mTOR-S6K pathway. Mucosal Immunol 8:80–93. doi:10.1038/mi.2014.44 24917457 PMC4263689

[68] Luu M, Pautz S, Kohl V, Singh R, Romero R, Lucas S, Hofmann J, Raifer H, Vachharajani N, Carrascosa LC, Lamp B, Nist A, Stiewe T, Shaul Y, Adhikary T, Zaiss MM, Lauth M, Steinhoff U, Visekruna A. 2019. The short-chain fatty acid pentanoate suppresses autoimmunity by modulating the metabolic-epigenetic crosstalk in lymphocytes. Nat Commun 10:760. doi:10.1038/s41467-019-08711-2 30770822 PMC6377655

[69] Plaizier JC, Mulligan FJ, Neville EW, Guan LL, Steele MA, Penner GB. 2022. Invited review: effect of subacute ruminal acidosis on gut health of dairy cows. J Dairy Sci 105:7141–7160. doi:10.3168/jds.2022-21960 35879171

[70] Cicero AFG, Fogacci F, Di Micoli V, Angeloni C, Giovannini M, Borghi C. 2023. Purine metabolism dysfunctions: experimental methods of detection and diagnostic potential. Int J Mol Sci 24:7027. doi:10.3390/ijms24087027 37108190 PMC10138451

[71] Zhang L, Na X, Lai B, Song Y, Wang H, Tan M. 2021. Effects of fluorescent carbon dots from the baked lamb on energy and lipid metabolism. Food Chem 338:127832. doi:10.1016/j.foodchem.2020.127832 32818868

[72] Petrus P, Cervantes M, Samad M, Sato T, Chao A, Sato S, Koronowski KB, Park G, Alam Y, Mejhert N, Seldin MM, Monroy Kuhn JM, Dyar KA, Lutter D, Baldi P, Kaiser P, Jang C, Sassone-Corsi P. 2022. Tryptophan metabolism is a physiological integrator regulating circadian rhythms. Mol Metab 64:101556. doi:10.1016/j.molmet.2022.101556 35914650 PMC9382333

[73] Davies SK, Ang JE, Revell VL, Holmes B, Mann A, Robertson FP, Cui N, Middleton B, Ackermann K, Kayser M, Thumser AE, Raynaud FI, Skene DJ. 2014. Effect of sleep deprivation on the human metabolome. Proc Natl Acad Sci USA 111:10761–10766. doi:10.1073/pnas.1402663111 25002497 PMC4115565

[74] Yang Z, Chen Z, Chen L, Xiong G. 2022. 5-Hydroxyindoleacetic acid, a new ligand for GPR35, plays an important role in inflammatory disease. Acta Biochim Biophys Sin (Shanghai) 55:169–171. doi:10.3724/abbs.2022198 36647721 PMC10157522

[75] Lopes JG, Sourjik V. 2018. Chemotaxis of Escherichia coli to major hormones and polyamines present in human gut. ISME J 12:2736–2747. doi:10.1038/s41396-018-0227-5 29995838 PMC6194112

[76] Hernández-Castellano LE, Hernandez LL, Bruckmaier RM. 2020. Review: endocrine pathways to regulate calcium homeostasis around parturition and the prevention of hypocalcemia in periparturient dairy cows. Animal 14:330–338. doi:10.1017/S1751731119001605 31337460

[77] Miletta MC, Petkovic V, Eblé A, Ammann RA, Flück CE, Mullis PE. 2014. Butyrate increases intracellular calcium levels and enhances growth hormone release from rat anterior pituitary cells via the G-protein-coupled receptors GPR41 and 43. PLoS One 9:e107388. doi:10.1371/journal.pone.0107388 25310566 PMC4195582

[78] Li QS, Wang R, Ma ZY, Zhang XM, Jiao JZ, Zhang ZG, Ungerfeld EM, Yi KL, Zhang BZ, Long L, Long Y, Tao Y, Huang T, Greening C, Tan ZL, Wang M. 2022. Dietary selection of metabolically distinct microorganisms drives hydrogen metabolism in ruminants. ISME J 16:2535–2546. doi:10.1038/s41396-022-01294-9 35931768 PMC9562222

[79] Fukumori R, Doi K, Mochizuki T, Oikawa S, Gondaira S, Iwasaki T, Izumi K. 2022. Sodium butyrate administration modulates the ruminal villus height, inflammation-related gene expression, and plasma hormones concentration in dry cows fed a high-fiber diet. Anim Sci J 93:e13791. doi:10.1111/asj.13791 36478496

[80] Klobucher KN, Badger R, Foxall T, Erickson PS. 2022. Short Communication: effect of sodium butyrate, monensin, and butyric acid on the viability of Eimeria bovis sporozoites and their degree of damage to a bovine epithelial cell line. J Anim Sci 100:skac360. doi:10.1093/jas/skac360 36315476 PMC9733496

[81] Jiang L, Wang J, Liu Z, Jiang A, Li S, Wu D, Zhang Y, Zhu X, Zhou E, Wei Z, Yang Z. 2020. Sodium butyrate alleviates lipopolysaccharide-induced inflammatory responses by down-regulation of NF-κB, NLRP3 signaling pathway, and activating histone acetylation in bovine macrophages. Front Vet Sci 7:579674. doi:10.3389/fvets.2020.579674 33251265 PMC7674777

